# Metabolic engineering of phosphite metabolism in *Synechococcus elongatus* PCC 7942 as an effective measure to control biological contaminants in outdoor raceway ponds

**DOI:** 10.1186/s13068-020-01759-z

**Published:** 2020-07-09

**Authors:** Sandra Isabel González-Morales, Navid Berenice Pacheco-Gutiérrez, Carlos A. Ramírez-Rodríguez, Alethia A. Brito-Bello, Priscila Estrella-Hernández, Luis Herrera-Estrella, Damar L. López-Arredondo

**Affiliations:** 1 StelaGenomics México, S de RL de CV, Av. Camino Real de Guanajuato s/n, Irapuato, 36821 Guanajuato, Mexico; 2 Laboratorio Nacional de Genómica para la Biodiversidad, Unidad de Genómica Avanzada del Centro de Investigación y de Estudios Avanzados del Instituto Politécnico Nacional, Km 9.6 carretera Irapuato León, Irapuato, 36500 Guanajuato, Mexico; 3 Institute of Genomics for Crop Abiotic Stress Tolerance, Texas Tech University, Lubbock, TX 79409 USA

**Keywords:** Phosphite metabolism, Outdoor cultivation, Phosphite oxidoreductase, Biological contamination, Selectable marker, *Synechococcus elongatus* PCC 7942

## Abstract

**Background:**

The use of cyanobacteria and microalgae as cell factories to produce biofuels and added-value bioproducts has received great attention during the last two decades. Important investments have been made by public and private sectors to develop this field. However, it has been a challenge to develop a viable and cost-effective platform for cultivation of cyanobacteria and microalgae under outdoor conditions. Dealing with contamination caused by bacteria, weedy algae/cyanobacteria and other organisms is a major constraint to establish effective cultivation processes.

**Results:**

Here, we describe the implementation in the cyanobacterium *Synechococcus elongatus* PCC 7942 of a phosphorus selective nutrition system to control biological contamination during cultivation. The system is based on metabolic engineering of *S. elongatus* to metabolize phosphite, a phosphorus source not normally metabolized by most organisms, by expressing a bacterial phosphite oxidoreductase (PtxD). Engineered *S. elongatus* strains expressing PtxD grow at a similar rate on media supplemented with phosphite as the non-transformed control supplemented with phosphate. We show that when grown in media containing phosphite as the sole phosphorus source in glass flasks, the engineered strain was able to grow and outcompete biological contaminants even when the system was intentionally inoculated with natural competitors isolated from an irrigation canal. The PtxD/phosphite system was successfully used for outdoor cultivation of engineered *S. elongatus* in 100-L cylindrical reactors and 1000-L raceway ponds, under non-axenic conditions and without the need of sterilizing containers and media. Finally, we also show that the PtxD/phosphite system can be used as selectable marker for *S. elongatus* PCC 7942 transgenic strains selection, eliminating the need of antibiotic resistance genes.

**Conclusions:**

Our results suggest that the PtxD/phosphite system is a stable and sufficiently robust strategy to control biological contaminants without the need of sterilization or other complex aseptic procedures. Our data show that the PtxD/phosphite system can be used as selectable marker and allows production of the cyanobacterium *S. elongatus* PCC 7942 in non-axenic outdoor reactors at lower cost, which in principle should be applicable to other cyanobacteria and microalgae engineered to metabolize phosphite.

## Background

Cyanobacteria are emerging as promising systems for biotechnological applications. They offer a number of advantages over other microorganisms, including rapid reproduction with conversion rates into biomass much higher than that of plants, as well as the possibility of photoautotrophic cultivation harvesting environmental CO_2_ and harnessing solar light energy [1]. Also, cyanobacteria do not compete for arable land with crop cultivation and some species can be cultivated using wastewater [2]. A number of cyanobacterial species have been explored not only as laboratory models, but also for development of platforms to produce ethanol, chemicals, high-value bioproducts, cosmetics, nutraceutics, and as biofertilizers in agriculture [3–5].

Although progress in developing genetic and genomic tools has been slow compared to that for bacteria or even plants, genetic manipulation of some cyanobacterial strains by classical genetic approaches, gene transfer, and genome editing-based techniques is now possible. Model cyanobacteria such as *Synechococcus elongatus* PCC 7942 and UTEX 2973, and *Synechocystis* sp. PC6803 have been engineered through different strategies to produce a number of chemicals such as isoprene, acetone and ethanol, offering new avenues to potentiate production of target compounds [6–8]. To have a significant economic, environmental, and social impact of these applications, the main challenge is to design production schemes that warrant biomass production of the desired strain at a cost-effective level using inexpensive reactors with low operation costs. To date, the most convenient and cost-effective type of reactor for cyanobacteria and microalgae cultivation are raceway ponds operated under outdoor conditions. However, in outdoor systems, strains are challenged by environmental conditions and are highly susceptible to contamination by other microorganisms, including bacteria, yeast, fungi, weed algae/cyanobacteria, and protozoans [9, 10]. Biological contaminants must be quickly and effectively controlled, as these organisms can rapidly cause complete loss of production batches. Cyanobacteria are competitive organisms and some fast-growing strains have been reported recently [9, 10]. However, under laboratory conditions, they display growth rates slower than that of many bacteria (i.e., doubling time of *S. elongatus* PCC 2973, *Synechococcus* PCC 7942, and *Synechocystis* PCC 6803, is 1.9, 4, and 7–10 h, respectively), representing a potential disadvantage if scaled-up under outdoor conditions. A similar situation is faced for microalgal strains which usually display even slower doubling times [11]. Thus, production costs become a major factor in selecting cyanobacterial versus bacterial as production systems.

Invasion of cyanobacterial cultures by contaminant organisms has long being recognized as a major constraint for large-scale cultivation, which occurs not only in open cultivation systems, but also on closed and hybrid systems that have been specially designed to decrease contamination risks [12, 13]. To control contamination, chemical treatments such as use of herbicides, antibiotics, detergents, hypochlorite, and phenol are often used [14, 15]. Moreover, in most cases sterilization of growth media and bioreactors must be implemented to maintain the desired monoculture. However, these practices substantially increase operating costs for closed or hybrid bioreactors and open ponds cannot be sterilized or kept under sterile conditions. A strategy to deal with contamination in outdoor open ponds is the use of selective culture environments such as high salt concentrations for halotolerant strains [12, 16, 17] or N-deprived media for N-fixing cyanobacteria [18]. Unfortunately, these practices are limited to a few cyanobacterial species and can also affect final product quality. An additional measure which may favor the establishment of the target strain in sequential open ponds of different sizes, is using high inoculum percentage, because opportunistic organisms, naturally present and more adapted to environmental conditions, may dominate the system more easily when the starter culture is small [19]. For production of bioproducts, deployment of the target cyanobacterium, native or engineered, should be ideally outdoor and with minimal external requirements.

Previously, we reported on the implementation of the metabolic engineering of *Chlamydomonas reinhardtii* to metabolize phosphite (Phi) as an effective strategy to control biological contamination. This was achieved by expressing a *ptxD* gene encoding a phosphite oxidoreductase (PtxD), that enable the engineered strains to metabolize Phi as the sole phosphorus (P) source, and therefore, outcompete contaminant organisms unable to use Phi as a P source [20]. This system allows the establishment of a highly selective environment by using Phi as a growth control agent and P source at the same time. More recently, the Phi metabolism was also established as a biocontainment strategy that allows the control of proliferation of genetically modified *S. elongatus* in case an accidental release to the environment, as well as a selectable marker for microalgae, cyanobacteria, and several plant species [21–25]. Biotechnological applications of the selective nutrition based on Phi and the *ptxD* gene for microalgae/cyanobacteria cultivation have been successful as a proof of concept using small volume closed or semi-closed systems [20, 21, 23, 26]. However, there are genuine concerns regarding the stability and robustness of the Phi metabolism trait for cultivation in larger scale reactors in outdoor conditions. Here, we report the metabolic engineering of *Synechococcus elongatus* PCC 7942 to assimilate Phi by expressing only a codon-optimized sequence of *ptxD*, without the need of a Phi-specific transporter. We also report the successful containment of contamination in the scaled cultivation of the engineered strain up to 1000-L raceway pond under outdoor non-axenic conditions. The engineered strain became the dominant species in mixed cultures without any additional measure to avoid contamination. Our results show that the capacity of metabolizing Phi provides a competitive advantage to the engineered strain that effectively prevents or severely limits invasion of open or closed culture systems by undesirable biological contaminant. We also show that the PtxD/Phi system can be used as a selectable marker in *S. elongatus* PCC 7942. This is the first report providing evidence of the stability and robustness of the PtxD/Phi technology for outdoor cultivation of the model cyanobacterium *S. elongatus*.

## Results

### *Synechococcus elongatus* strains expressing *ptxD* are able to use phosphite as the sole phosphorus source

We hypothesized that a metabolic advantage to outcompete contaminant organisms can be provided to *S. elongatus* PCC 7942 (SeWT) strain by expressing the *ptxD* gene from *P. stutzeri* WM88 and using Phi as the sole P source. We first studied the effect of different concentrations of Phi (0.1, 0.2, 0.8, 1, 1.8, 2 mM) on the growth of SeWT in liquid media as compared to growth in media supplemented with phosphate (Pi), the normal P source, or lacking a P source, as positive and negative controls, respectively. When SeWT cells were inoculated in control media devoid of a P source, no increase in cell density was observed (Additional file 1: Figure S1); while as expected, in media supplemented with Pi the SeWT culture displayed rapid growth reaching a cell density higher than 1.6 × 10^9^ cells/mL after 6 days of cultivation (Additional file 1: Figure S1). By contrast, no increase in SeWT cell density at any Phi concentration was detected. These results showed that SeWT is unable to metabolize Phi.

**Fig. 1:**
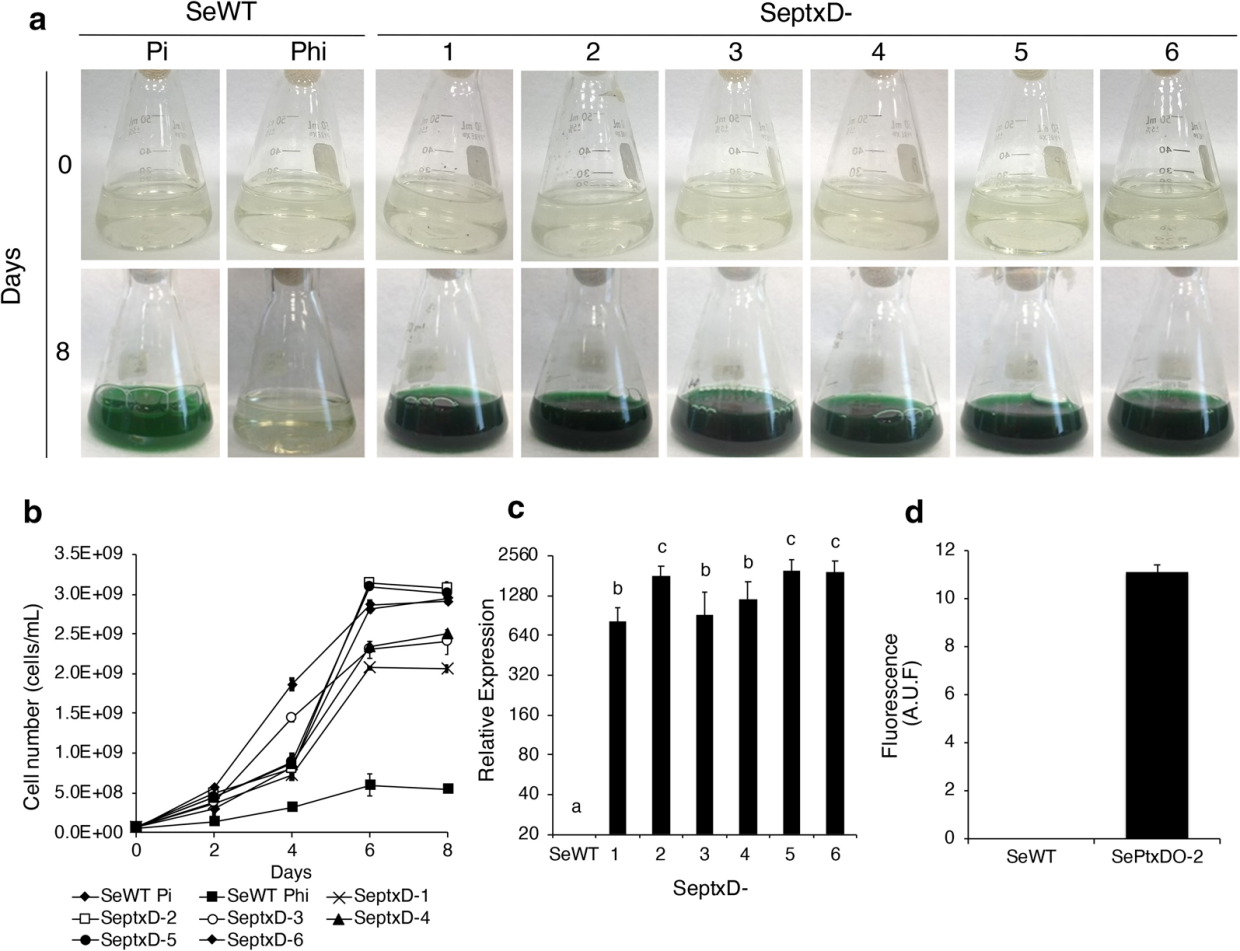
Characterization and growth of *Synechococcus elongatus* transgenic strains in media with phosphite. **a** Transgenic strains SeptxD-1 to -6 were grown in BG-11 media supplemented with 1.8 mM of phosphite (Phi) as phosphorus source. *S. elongatus* PCC 7942 (SeWT) was cultured in media with Phi and phosphate (Pi, 0.2 mM) as controls. Cultures were photographed at 0 and 8 days after inoculation. **b** Cell number was determined every 2 day during 8 days. **c** Relative expression of the *ptxD* gene in *S. elongatus* transgenic strains (SeptxD-1 to -6). *S. elongatus* PCC 7942 (SeWT) strain was used as negative control; *SerpoD* and *SernpB* genes were used as housekeeping genes. **d** PtxD enzymatic activity expressed as Arbitrary Units of Fluorescence (A.U.F.) determined in SeptxD-2 and SeWT as negative control. Values are the mean of three replicates ± SE (n = 3). Different letters indicate significant differences (ANOVA test, *P *< 0.05)

As a next step, we designed a DNA construct harboring a codon-optimized version of the *ptxD* gene based on *S. elongatus* codon usage, under control of the *psbAI* constitutive promoter [27] (see Methods section). This construct was inserted into a plasmid vector also containing a *pSp::aadA2* gene cassette for resistance to spectinomycin and used for the genetic transformation of SeWT using homologous recombination into the neutral site 1 (NS1). Hundreds of independent colonies resistant to spectinomycin were obtained in each transformation experiment. Six fast-growing colonies (SeptxD-1 to 6) were selected to test their capacity to use Phi as their sole P source. The six selected transformed strains were directly cultured in liquid media containing 1.8 mM Phi as the sole P source. After 6 days of cultivation we observed that the six *ptxD*-transgenic strains were capable of growing using Phi as a sole P source, to a similar extent to that observed for SeWT in media supplemented with Pi as P source (Fig. 1a). However, when we determined cell density (cells/mL), some quantitative growth differences were observed between the different *ptxD*-transgenic strains (Fig. 1b). Strains SeptxD-2, -5, and -6 reached a density of over 3 × 10^9^ cells/mL in media supplemented with Phi, which was similar or slightly better than that observed for the SeWT using Pi (2.9 × 10^9^ cells/mL), whereas strains SeptxD-1, -3 and -4 reached slightly lower cell densities (~ 2.3 × 10^9^ cells/mL) (Fig. 1b). The expression level of the *ptxD* gene was also determined by RT-qPCR analysis. The *ptxD* transcript was detected in all the engineered strains, being higher in strains SeptxD-2, -5, and -6 than in strains SeptxD-1, -3 and -4. *ptxD* transcript was not detected in the WT untransformed control (Fig. 1c). The higher level of *ptxD* transcript in strains 2, 5, and 6 corresponds with their better growth in Phi media when compared with strains 1, 3, and 4. The activity of the PtxD enzyme in one of the transgenics trains, SeptxD-2, was also confirmed by an optimized fluorometric protocol (Fig. 1d). As expected, no PtxD activity was observed in the WT control, whereas the signal was clearly detected in the transgenic strain (Fig. 1d). The correct integration of the expression cassette in the six engineered strains initially tested, was confirmed by PCR using primers specific for the NS1 and the *ptxD* gene, and the fragments verified by Sanger sequencing (Additional file 1: Figure S2).

To determine the concentration of Phi that sustained optimal growth of the engineered strains, we tested the growth of SeptxD-2 in media supplemented with 0.1, 0.8, 1.5, 1.8, 2.5, 3, and 5 mM Phi as a sole P source. We observed that SeptxD-2 was able to grow in almost all tested Phi concentrations, excepting in 0.1 mM, but that optimal growth was achieved at 1.8 mM Phi (Additional file 1: Figure S3). The poor growth of SeptxD-2 at 0.1 mM is probably due to an insufficient amount of P to sustain the growth of the transgenic line whereas high Phi concentrations (2.5–5 mM) may exert an inhibitory effect. The slight growth of SeWT in media with Phi observed in some of these experiments was probably due to the use of an inoculum that came from containing media containing 0.2 mM Pi (Fig. 1b), which accumulate Pi reserves that can be used to sustain initial growth in media lacking a P source or in the presence of Phi [28].

### SeptxD-2 transgenic strain is able to overcome competition from a model microalga and naturally occurring contaminants

**Fig. 2:**
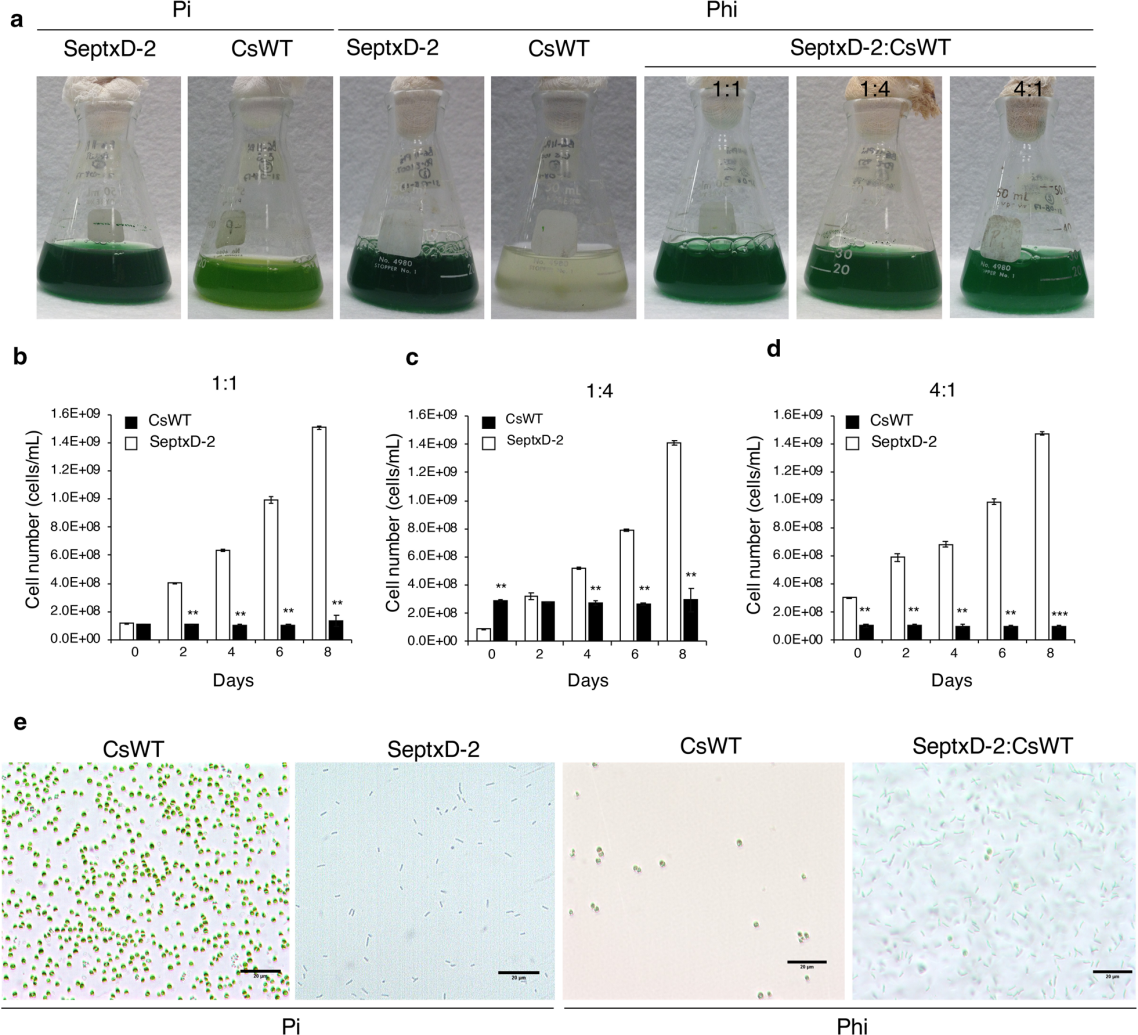
Growth competition experiments between SeptxD-2 and *Chlorella sorokiniana* using phosphite as the only phosphorus source. **a***S. elongatus* transgenic strain SeptxD-2 and *Chlorella sorokiniana* UTEX 1230 (CsWT) were grown as monocultures in BG-11 media with phosphate (Pi, 0.2 mM) as the phosphorus (P) source, and as monocultures and mixed cultures in three different inocula proportions SeptxD-2:CsWT (1:1, 1:4, and 4:1) in BG-11 media with 1.8 mM phosphite (Phi) as P source. Cultures were photographed 8 days after inoculation. Cell number (cells/mL) was determined for the experiments with different proportions, **b** 1:1, **c** 1:4, **d** 4:1, every 2 days. **e** Cultures on (**a**, **b**) were analyzed by light microscopy 8 days after inoculation. Black bar corresponds to 20 μm. Values are the mean of three replicates ± SD. Bars with asterisks are significantly different from the control (Student’s *t* test, *P < 0.05, **P < 0.01, ***P < 0.0001)

The capacity of the strain of interest to outcompete other microalgae/cyanobacteria is crucial to obtaining the desired product in outdoor conditions. Cultivation media provide all the essential nutrients (i.e., P) that favor cell viability and facilitate reproduction of the strains, allowing maximum production of biomass under optimal conditions. Therefore, all microalgae/cyanobacteria naturally present in the environment will also compete for P and other resources with the desired strain, which is crucial at early stages of cultivation. To test whether Phi metabolism provides *ptxD*-transgenic strains a growth advantage to compete against contaminant organisms, we performed competition experiments between SeptxD-2 with a fast-growing microalga. We selected *Chlorella sorokiniana* (CsWT) as a competitor microalga because displays a growth rate similar to that of *S. elongates* in BG-11 media and has a clearly distinguishable morphology from that of the cyanobacterium [29]. Since SeWT is rod shaped and CsWT is larger and spherical, each cell type is easily identified by microscopy. In these experiments, we included monocultures of each strain under Pi and Phi treatment, as well as mixed cultures with three different proportions of the inocula, 1:1, 1:4 and 4:1 (SeptxD-2:CsWT) (Fig. 2). When the strains were grown as monocultures in media with Pi, we observed similar growth rate of both species reaching a density of about 1.2 × 10^9^ cells/mL after 8 days of cultivation (Fig. 2a, Additional file 1: Figure S4a, b). However, when cultured in media with Phi, only SeptxD-2 rapidly proliferated at about 20% higher growth than that obtained in media supplemented with Pi, whereas CsWT was unable to grow and its cell density did not increase with the time (Fig. 2a, Additional file 1: Figure S4). As expected, when the transgenic strain was confronted with CsWT and cultivated in Phi media, SeptxD-2 rapidly outgrew the competitor microalga at all inoculation proportions (1:1, 1:4, and 4:1). We did not observe a significant increase in CsWT cell density during the 8 days of the competition experiment (Fig. 2a–d). Growth of SeptxD-2 increased substantially from the first day of cultivation, even when the initial inoculum of CsWT competitor was fourfold higher than the *ptxD*-engineered strain (Fig. 2c). Under this condition, SeptxD-2 reached 1.4 × 10^9^ cells/mL after 8 days of cultivation, which was substantially superior to that displayed by CsWT under the same condition (Fig. 2c). Differences in growth were easily visible under a microscope. Only spherical CsWT or rod-shaped SeptxD-2 cells were observed when grown as monocultures using Pi (Fig. 2e), whereas in the competition experiments in Phi media SeptxD-2 cells predominated over the competitor (Fig. 2e).

**Fig. 3:**
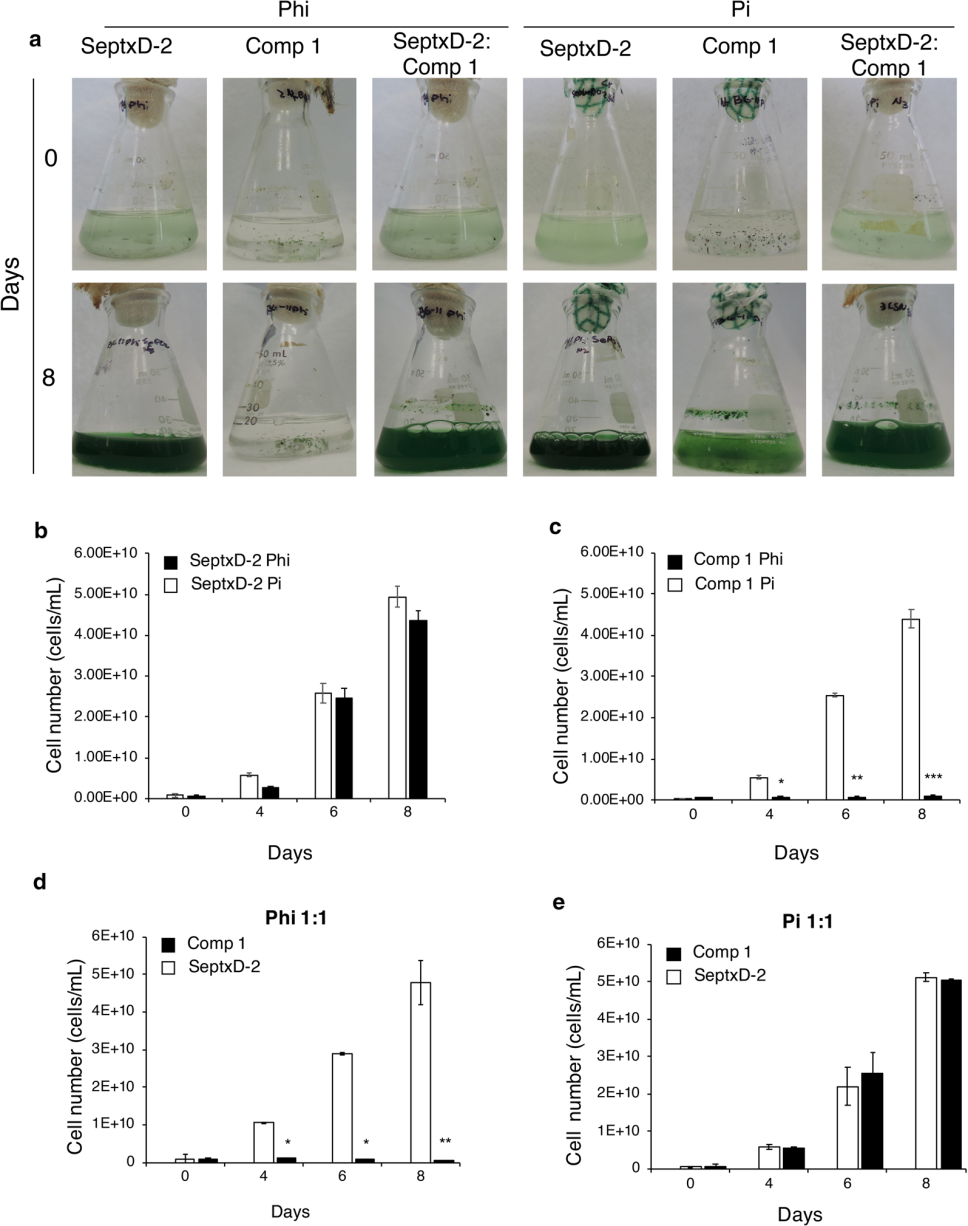
Growth competition experiments between SeptxD-2 and a natural competitor using phosphite as the only phosphorus source. **a***S. elongatus* transgenic strain (SeptxD-2) and a natural competitor (Comp 1) were grown in monocultures and mixed cultures using BG-11 medium supplement with 0.2 mM phosphate (Pi) or 1.8 mM phosphite (Phi) as phosphorus source. Cultures were photographed 8 days after the inoculation. Cell number (cells/mL) was determined for the monocultures of SeptxD-2 (**b**), and the natural competitor 1 (Comp 1) (**c**), and for the mixed cultures under Phi (**d**) and Pi (**e**) treatments. Values are the mean of three replicates ± SD. Bars with asterisks are significantly different from the control (Student’s *t* test, *P < 0.05, **P < 0.01, ***P < 0.0001)

To test whether the transgenic strain is also able to outcompete other more natural competitors from the environment, we carried out similar experiments as described before but using competitors that we isolated from stationary irrigation canals located near our facilities. We collected a number of samples from two different water canals that feed agricultural fields in the Bajio region (Guanajuato, México, see Methods section). Algal bloom frequently occurs in these water bodies due to nutrient run-off from crop fertilization. From these samples, we obtained some consortia (denominated competitor, Comp) that grew well in the same media used for *S. elongatus*. To have an idea of the nature of the wild organisms present in the isolates, the 16S rRNA of Comp 1 was sequenced. The results indicated the presence of members of the genera Nostoc and Allinostoc (Additional file 2: Table S1). Two of the consortia with predominant species of spherical shaped cells, Comp 1 and Comp 2, were used to challenge axenic SeptxD-2 using 1:1 or 1:4 (SeptxD-2:Comp) inocula proportions. After 8 days of growth as monoculture, Comp 1 and SeptxD-2 displayed vigorous growth in media supplemented with Pi as P source reaching cell densities of 4.3 × 10^10^ and 4.9 × 10^10^ cells/mL, respectively (Fig. 3a–c). As previously observed, SeptxD-2 was also able to sustain rapid growth in media supplemented with Phi as a sole P source reaching a cell density (4.36 × 10^10^ cells/mL), whereas no detectable growth was registered in media supplemented with Phi for the microorganisms present in the Comp 1 consortium (Fig. 3a–c). When SeptxD-2 was challenged by Comp 1 as a competitor in Phi media, SeptxD-2 sustained normal growth, achieving a density of 4.79 × 10^10^ cells/mL, whereas Comp 1 cells did not grow (Fig. 3d, Additional file 1: Figure S4). In media supplemented with Pi, both SeptxD-2 and Comp 1 reached cell densities similar to that observed when cultivated as monocultures (Fig. 3e; Additional file 1: Figure S5). Interestingly, for the case of Comp 2, we observed less growth when cultivated as monoculture in Pi media (Additional file 1: Figure S6c), which was enhanced when in competition with SeptxD-2 under the same condition (Additional file 1: Figure S6e). However, when in competition with SeptxD-2 in Phi media, growth of Comp 2 was effectively controlled by Phi (Additional file 1: Figure S6d). These data suggest that the PtxD/Phi system is effective to control contamination by organisms present in the environment that could act as a natural contaminant and could compromise growth of microalga/cyanobacterium of interest in raceway ponds. Therefore, the PtxD/Phi system seems suitable for outdoor cultivation under non-sterile conditions.

### *ptxD* together with phosphite is effective to control contaminants in outdoor cultivation systems

Scaling-up outdoor cultivation of cyanobacteria and microalgae is a prerequisite to implement commercially viable processes to produce biomass or bioactive compounds. In order to validate the effectiveness and robustness of the PtxD/Phi system in outdoor open conditions without the use of sterilization to prepare media or bioreactors, we implemented a cultivation process of the SeptxD-2 strain to scale up to an outdoor 1000-L raceway pond (Additional file 1: Figure S7). The first steps of propagation, from Petri dishes to 1-L glass bottles, of SeptxD-2 and SeWT were carried out under sterile conditions in the laboratory. Cultures of SeWT and SeptxD-2 were initiated by scraping a portion of the culture from solid media in a Petri dish that was suspended in 5 mL of liquid media to inoculate a 50-mL flask with 25 mL of media; 20 mL of this culture were used to inoculate 700 mL of media in 1-L glass bottles, which was then used to inoculate a 7-L home-designed cylindrical bioreactor. Since the percentage of inoculum seems to be crucial to start a rapidly growing culture, we first tested 3, 5, and 7% inoculum of the transgenic strain. We observed that 3 or 5% SeptxD-2 inoculum was insufficient to establish a successful culture and 7% inoculum led to a final high cell density (Additional file 1: Figure S8). Using 7% inoculum of SeWT and SeptxD-2 cultivated in sterile Pi and non-sterile Phi media, respectively, we observed that the transgenic strain produced a little higher biomass (0.53 g/L) than the control (0.43 g/L) (Additional file 1: Figure S9). Cultures produced in 7-L cylindrical bioreactors were then used to inoculate a home-designed 100-L cylindrical bioreactors. To determine the percentage of inoculum required to establish a successful 100-L culture, we again tested 3, 5, 7, and 10% inocula of SeWT and SeptxD-2 cultivated in non-sterile Pi and Phi media, respectively, in a 100-L cylindrical open reactor. It is important to note that for this step the reactor and media to grow SeWT, as well as SeptxD-2, were not sterilized prior to inoculation. After 8 days of inoculation, we observed that none of the amounts of SeWT inoculum allowed the establishment of a successful culture in the 100-L reactor and because of a slightly milky color in the media apparently only non-photosynthetic organisms grew in Pi media (Additional file 1: Figure S10). In the case of SeptxD-2, the cyanobacterium did not proliferate using 3 and 5% inoculum, and little bacterial proliferation was observed as the media remained nearly transparent (Additional file 1: Figure S10). A successful culture of SeptxD-2 in 100-L reactor was established using 7 and 10% inoculum, with slight differences in growth curves (Additional file 1: Figure S10); suggesting that 7% inoculum can be used to decrease operation costs (Additional file 1: Figure S11).

**Fig. 4:**
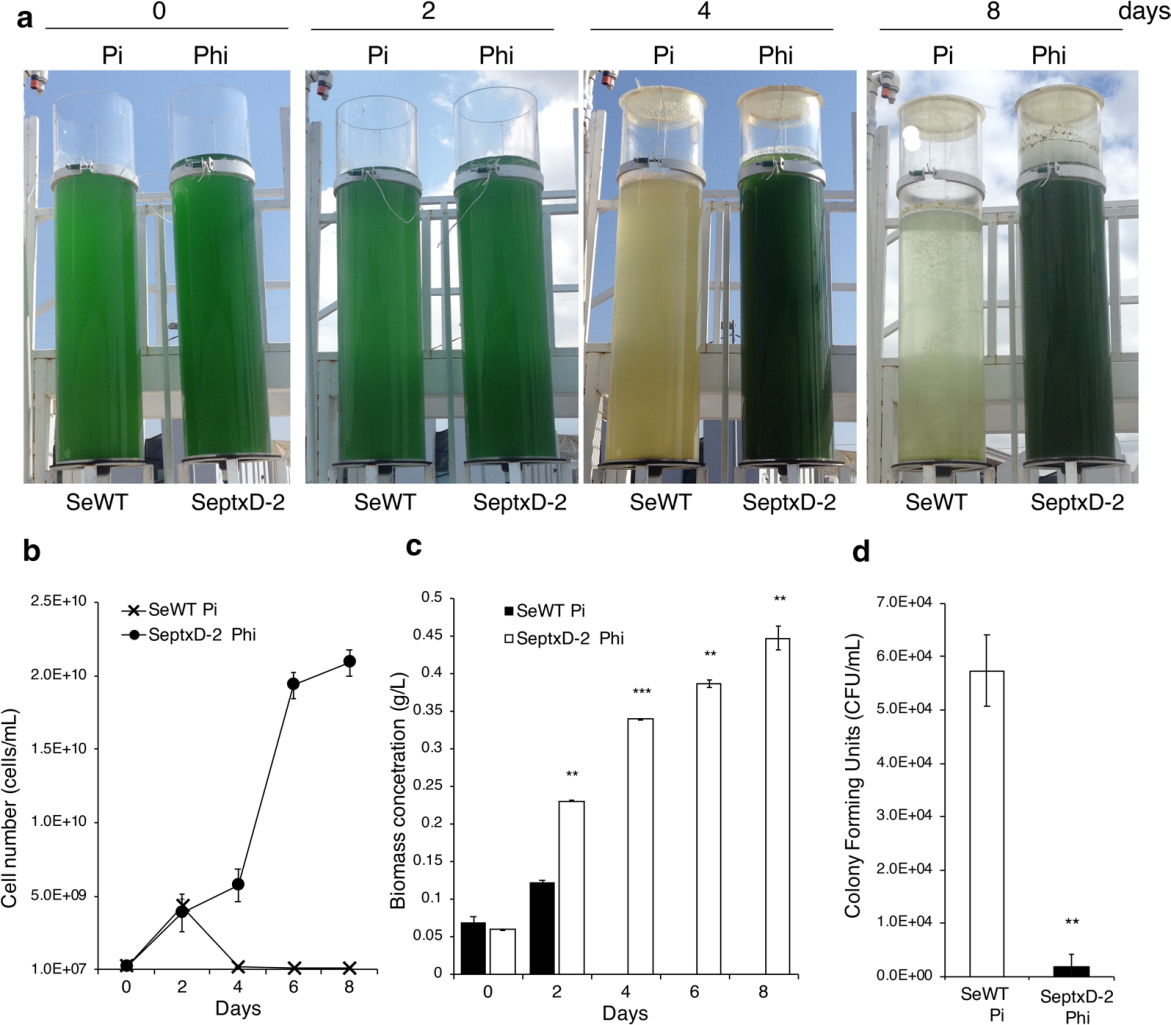
SeptxD-2 cultivation in outdoor cylindrical reactors using phosphite as phosphorus source under non-sterile conditions. **a***S. elongatu*s PCC 7942 (SeWT) and SeptxD-2 transgenic strain were grown in BG-11 media prepared with industrial grade reagents and supplemented with 1.8 mM phosphite (Phi) and 0.2 mM phosphate (Pi) as P source, respectively. **b** Cell number (cells/mL) and **c** biomass were determined every 2 days during the experiments. **d** Colony Forming Units (CFU/mL) derived from SeptxD-2 and SeWT cultures. Cultures were performed using 7% (v/v) inoculum and non-sterile 100-L cylindrical reactors bubbled with air outdoor. Values are the mean ± SD of three replicates. Bars with asterisks are significantly different from the control (Student’s *t* test, *P < 0.05, **P < 0.01, ***P < 0.0001). Photographs correspond to experiments carried out during summer, 2017

**Fig. 5:**
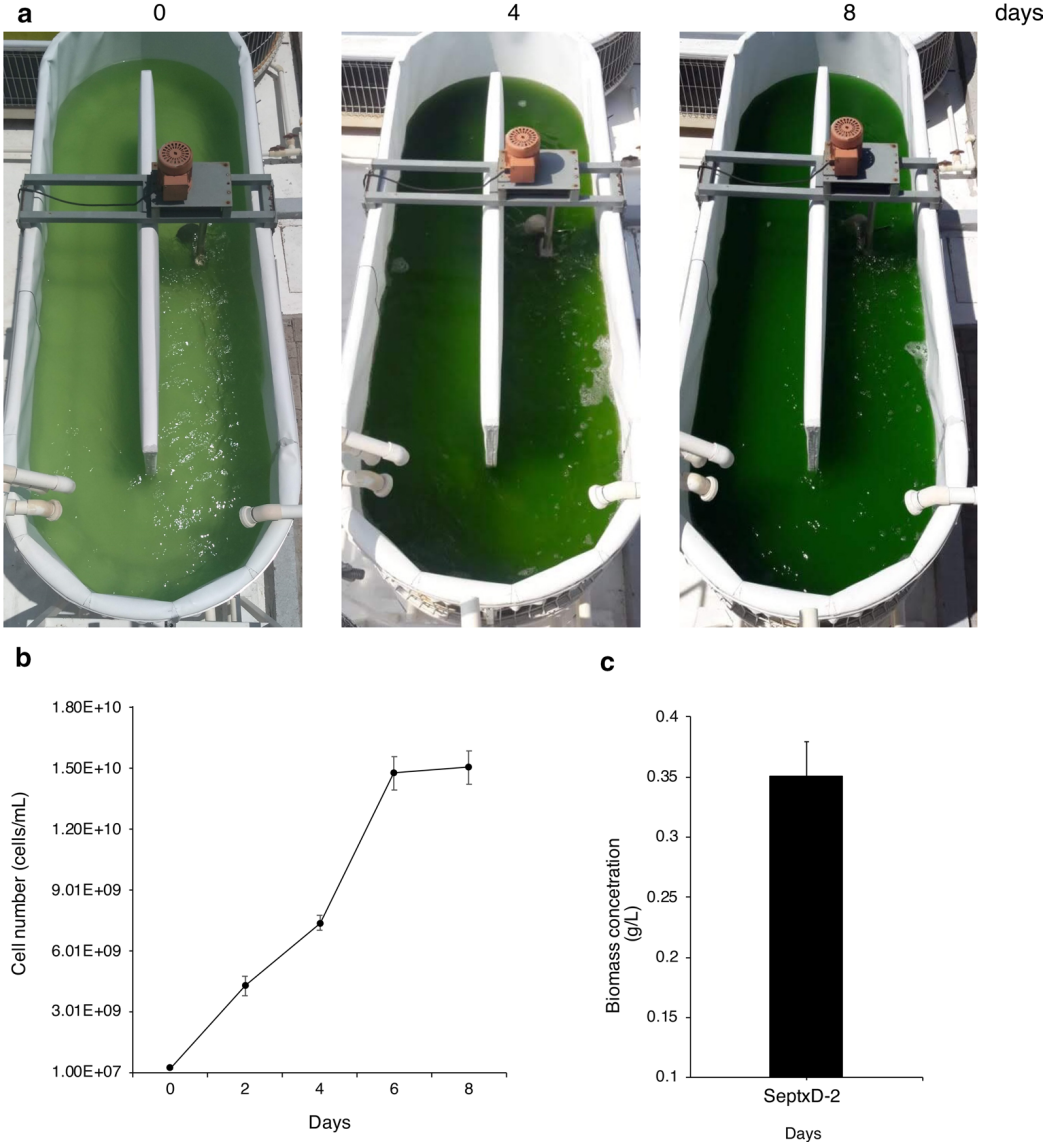
SeptxD-2 cultivation in raceways ponds using phosphite as phosphorus source under non-sterile conditions. **a** SeptxD-2 transgenic strain was grown in BG-11 media prepared with industrial grade reagents and supplemented 1.8 mM of phosphite (Phi) as P source in 1000-L raceway ponds in non-sterile, outdoor conditions. **b** Cell number (cells/mL) and **c** biomass were determined every 2 days during the experiments. Values are the mean of three replicates ± SD (Student’s *t* test, *P < 0.05, **P < 0.01, ***P < 0.0001). Photographs correspond to experiments carried out during summer, 2019

To further carry out scaling-up *S. elongatus* into an outdoor 1000-L raceway pond, cultures in 7 and 100-L cylindrical bioreactors were sequentially prepared using 7% inoculum and used to inoculate 1000-L raceway ponds outdoor under non-sterile conditions and without the use any other agent to control contamination. During outdoor experiments, we monitored solar irradiance, maximum and minimum environmental temperature, culture pH and temperature, and cell number (cells/mL). During summer experiments in 2017, 2 days after inoculation of SeWT strain in 100-L cylindrical bioreactors with Pi media, the culture began to decline and completely collapsed on day 4 (Fig. 4a, b), whereas SeptxD-2 grew normally reaching a cell density of 2 × 10^10^ cells/mL and a biomass production of 0.44 g/L, after 8 days (Fig. 4a, b). When SeptxD-2 was inoculated in the 1000-L open raceway pond, growth of the transgenic strain was stable during the timeframe of the experiment and reached 1.5 × 10^10^ cells/mL allowing a biomass production of about 0.35 g/L (Fig. 5a–c). Temperature and pH of the media, and environmental temperature and solar irradiance during the process were relatively stable and only slight changes were detected over the timeframe of the experiments (Additional file 2: Table S2). Similar results were observed in experiments performed during different seasons of different years (fall 2016, summer 2019) (Additional file 1: Figure S11; Additional file 2: Tables S3 and S4).

To monitor presence of contaminants in the cultures of SeWT and SeptxD-2 in 100-L cylindrical reactors outdoor, we took samples and determined colony forming units (CFU) of potential bacterial contaminants using Luria–Bertani (LB) rich medium, commonly used to grow bacteria. We observed that at 6 days after starting the experiment, CFU increased in media supplemented with Pi, where SeWT was growing, reaching CFU/mL of 5.7 × 10^4^ (Fig. 4d, Additional file 1: Figure S12). By contrast, in SeptxD-2 culture using media supplemented with Phi, contamination was several orders of magnitude lower (< 10^2^ CFUs/mL) than in media supplemented with Pi (Fig. 4d; Additional file 1: Figure S12). Our data suggest the PtxD/Phi system is stable under varying environmental conditions and robust enough to provide a competitive advantage to the strain of interest and to control external contamination in open reactors of at least 1000 L. Therefore, the system has the potential for large-scale production of *S. elongatus* and other species to produce lipids for biofuels or other bioactive compounds.

To comply with biosafety measures in Mexico, we monitored the potential dispersion of the transgenic strain into the environment and used an arrangement of traps in which 1200-L capacity plastic tanks were filled with 500 L of tap water supplemented with BG-11 media (see Methods section; Additional file 1: Figure S13). The tanks were placed north, south, and east from the cultivated open pond. In east direction, three traps were placed 3, 6, and 28 m from the raceway facility, whereas in the north and south, only a single tank was placed at 1.5 m from the cultivation place (Additional file 1: Figure S13). Samples were taken three times a week and analyzed by PCR and RT-qPCR to detect presence of the *ptxD* gene. No positive signal was detected in any of the samples collected from the different traps (Additional file 1: Figure S13), suggesting that no dispersion of the engineered strain occurs during the timeframe of these experiments.

### PtxD and phosphite can be used as selectable marker in *S. elongatus* PCC 7942

**Fig. 6:**
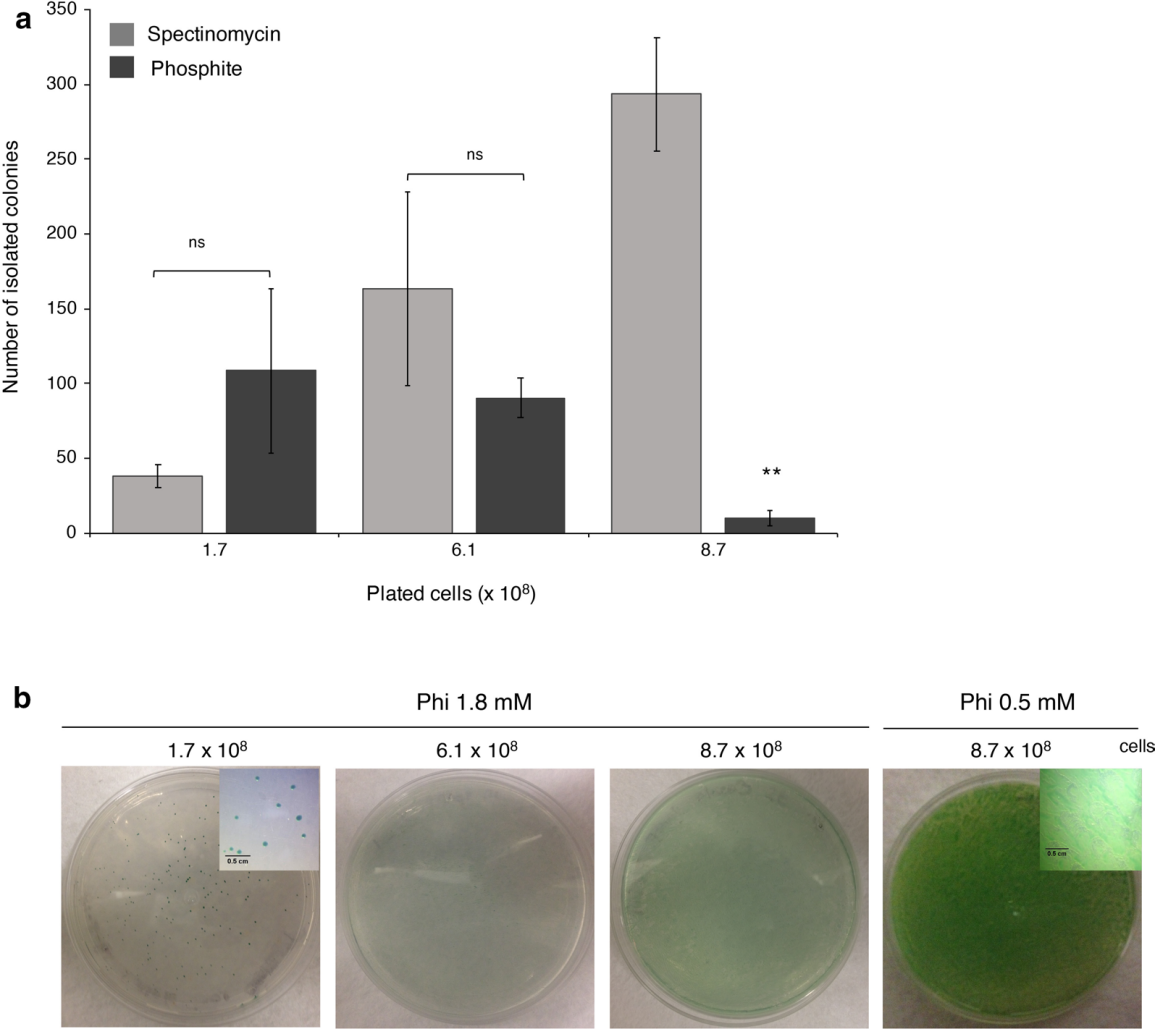
The PtxD/Phi system can be used as a selectable marker in *S. elongatus* PCC 7942. **a** Number of isolated colonies obtained 15 days after genetic transformation with *psbAI::SeptxD* construct. Three different amounts of cells (1.7, 6.1, and 8.7 × 10^8^) were spread onto agar plates with BG-11 medium supplemented with 1.8 mM Phi or 100 μg/mL spectinomycin. **b** Photographs of the agar plates were taken 15 days after cells were spread. A close-up view of the colonies is presented in the upright corner of the pictures. Values are the mean of three independent experiments with multiple plates each ± SD. Bars with asterisks are significantly different from spectinomycin selective condition (Student’s *t* test, *P < 0.05, **P < 0.01, ***P < 0.0001; ns, no significant differences)

Previous works demonstrated that the PtxD/Phi system can be used as an effective alternative selectable marker in *Synechococcus sp.* PCC 7002 [22]. To test whether the system can be also implemented in *S. elongatus* PCC 7942, an aliquot of cells (8.7 × 10^8^ cells/mL) were naturally transformed with the psyn_6_PtxDopt vector and spread directly onto agar plates with 1.8 mM Phi as sole P source, that resulted a very effective Phi concentration to suppress cell growth (Additional file 1: Figure S1). After 15 days, hundreds of green isolated colonies emerged, similar to spectinomycin selection (Fig. 6; Additional file 1: Figure S14). However, selection and isolation of Phi-resistant colonies was more effective when low amounts of cells were spread (i.e., 1.7 × 10^8^ cells/mL) per plate, because when the complete recombined aliquots were plated, according to the standard recommended protocol, a lawn of the cyanobacterium was observed and, thus, a poor efficiency of colony formation (Fig. 6). The use of lower Phi concentration (0.5 mM) failed to select transgenics (Fig. 6). Eleven colonies were randomly selected for PCR analysis to detect the *ptxD* gene and tested for growth using Phi. All of them were able to grow using 0.8 and 1.8 mM Phi and found PCR positive (Additional file 1: Figure S15). Thus, this system can be used to select colonies of *S. elongatus* PCC 7942 in which the desired heterologous genes have been integrated and are functional.

## Discussion

Biological contamination has been recognized by the NAAB (National Alliance for Advanced Biofuels and Bioproducts) and the DOE ASP (Aquatic Species Program) as one the most prevalent issues for high-scale cultivation of microalgae and cyanobacteria using different types of bioreactors [30, 31]. Strategies such as computerized methods, use of chemicals, and high-throughput sequencing for crop protection are thus highly recommended to maintain monocultures. Adopting these measures is critical when open ponds are used, as this type of bioreactor typically experiences frequent contamination that produce culture crash events. Therefore, due to the lack of effective, simple, and low-cost alternatives generally applicable for controlling biological contaminants, the recommendation is to search for more competitive local strains displaying the desired phenotypes over the use of elite and model strains. Although in some instances this is feasible, there may be the potential disadvantages of the lack of molecular tools and methods for improvement of wild species, which could hamper their exploitation as cell factories.

Here, we describe the efficacy of the phosphite oxidoreductase (PtxD)/phosphite (Phi) system to control biological contaminants in a scale of up to a 1000-L raceway pond reactor using the model cyanobacterium *Synechococcus elongatus*. The PtxD/Phi system was an effective tool to control biological contamination during the timeframe of the experiments by providing *S. elongatus* the capacity of metabolizing Phi as P source. In our experiments, we challenged the transgenic strain with the microalga *Chlorella sorokiniana* and two microbial consortia isolated from a close location. *S. elongatus* capable of metabolizing Phi was able to grow using Phi as its sole P source, outcompeting intentionally inoculated or natural contaminants present when using non-sterile outdoor conditions. Our research group originally developed the PtxD/Phi system for the control of weeds in agriculture [32, 33] and later proved it to also be effective for the control of biological contaminants in the cultivation of microalgae [20]. More recently, other research groups reported implementation of the PtxD/Phi system in model cyanobacteria, such as *Synechocystis sp*. PC6803, *S. elongatus* PCC 7942, *Synechococcus sp.* PCC 7002 [21, 22, 26]. Furthermore, the system was also refined to develop biocontainment strategies through more complex molecular strategies [21–23]. Previous works with *Synechocystis sp*. PC6803 and *S. elongatus* PCC 7942 proposed using a Phi-specific transporter system, in addition to the *ptxD* gene, as an essential component for successful implementation of the Phi metabolism. This is because the *ptxD*-expressing strains were unable to grow in media with low Phi concentrations as the P source and the WT strains seem to be unable to take up Phi [21, 26]. Here, we were able to successfully implement the Phi metabolism in *S. elongatus* PCC 7942 by expressing only a codon-optimized version of the *ptxD* gene, without the need of expressing an additional Phi-specific transporter. We observed that Phi concentrations below 0.8 mM are insufficient to sustain normal growth of the transgenic strains; and, that the most effective Phi concentration to support P nutrition is 1.8 mM. Similar results were reported previously for *ptxD*-expressing *Synechococcus sp*. PCC 7002 strains [22]. Engineered strains were able to use Phi without the need of a Phi-transporter; but, displayed restricted growth under 0.37 mM Phi, which improved under higher Phi concentrations and neared Pi culture normal growth at 7.4 mM Phi [22]. Motomura et al. [21] reported the use of 0.2 mM Phi in their experiments, in which the WT strain displayed slight growth. The engineered strains, harboring both the *ptxD* gene and the Phi-transporter genes, showed also a reduced growth using Phi compared to the WT using Pi, which was attributed to the type of transporter they expressed (HtxBCDE), that has an extremely low affinity for phosphite (*K*_*d*_ > 10 mM) [34].

The mechanism of transport and metabolism of Phi have been widely studied in *P. stutzeri* WM88, in which Phi is taken up via the PtxABC transporter and then oxidized into Pi by the PtxD oxidoreductase [34, 35]. Some reports suggest that certain marine cyanobacteria are able to take up and metabolize Phi as P source and Phi metabolism-related genes may be widely present in marine environments [26, 36, 37]. To our knowledge, this has been experimentally validated only for *Prochlorococcus* MIT930 and *Trichodesmium erythraeum* IMS101, which are capable of using Phi as P source and possesses a functional ptxABCD operon [26, 37]. However, no Phi transporters have been experimentally identified to date in many other microalgae or cyanobacteria, including *S. elongatus* PCC 7942 and *Synechococcus sp.* PCC 7002, and efforts to identify them using genomic information available have been also unsuccessful [21, 22, 38]. Although the physicochemical properties of Phi and Pi are distinct, it has been proposed that due to their structural similarities, Phi enters plant and microalgae cells using the same protein that transport Pi into the cell [36, 39, 40]. The reason behind higher Phi concentration than that of Pi required to provide optimal P nutrition to cyanobacterium expressing PtxD could be that the Pi transporters have lower affinity for Phi than Pi. In plants, two types of Pi transporters exist, low-affinity and high-affinity transporters, the latter operating when external concentrations of Pi are below 100 μM and the former when concentrations of Pi are above 300 μM [41, 42]. Since *ptxD*-transgenic plants sustain normal growth when supplied with 100 μM Phi, it is likely that both high and low-affinity Pi plant transporters are capable of efficiently transporting Phi. Therefore, it is also possible that only low-affinity, not high-affinity, Pi transporters are able to transport Phi into cyanobacterium cells. In this context, the fact that *ptxD*-expressing *Synechococcus* strains do not require a Phi-specific transporter to take up Phi and display normal growth only under high Phi concentrations, is a potential advantage for outdoor cultivation over a system dependent on the Phi-transporter. Further research is needed to elucidate Phi transport in cyanobacteria.

To date, the potential of the Phi-based system to control contaminations had only been tested using small volumes of growth media in Petri dishes, laboratory glassware, and small closed bioreactors [20–22, 26, 43]. Here we described the scaled-up cultivation process of a PtxD-engineered *S. elongatus* strains under outdoor conditions, using non-sterile 100-L cylindrical bioreactors and 1000-L open raceway ponds without any measure to control contaminants. *ptxD*-expressing culture was able to perform normally outdoor, whereas the WT culture collapsed due contamination 4 days after inoculation. SeptxD-2 displayed normal tolerance to varying environmental temperature, solar irradiance, and pH, during cultivation in a 1000-L outdoor raceway pond, suggesting that metabolism of Phi has no negative effect on the fitness of engineered *S. elongatus*. Moreover, we observed clear control of contamination as minimal presence of bacteria, rotifers, grazers, fungi or ciliates were observed.

Highly variable biomass concentration for different microalgae strains has been reported previously using different outdoor cultivation systems (polybags, stock ponds, and raceways). For instance, using stock ponds, biomass concentration of nine different strains ranged from 0.162 to 0.914 g/L [44], but apparently due photo-bleaching, only two of these strains, *Scenedesmus dimorphus* (UTEX 1237) and *Nannochloropsis salina* (NCMA 1776), were able to display consistent growth in raceways ponds [44]. We obtained *S. elongatus* biomass concentration of 0.44 and 0.35 g/L in 100-L cylindrical bioreactors and in 1000-L open ponds, respectively. Therefore, the biomass production we achieved using the PtxD/Phi system is an intermediate value in this range, but achieved in raceway pond rather than in a stock pond. Moreover, authors often report culture crashes due to contamination [44–49], which was not the case when using the PtxD/Phi system.

Sterilization of growth media using a micro filter cartridge and of tubing by washing with 95% ethanol for 5 min are commonly applied measures to prevent contamination for outdoor cultivation in polybags, stock ponds, and raceways [44]. Our results suggest that one of the major advantages of using Phi-metabolizing strains is the possibility of avoiding all these preventive measures, as the system does not require sterilizing growth media, reactors, tools, and piping. Thus, reducing one of the major operating costs for large closed and open bioreactors [20, 21, 23]. Therefore, the system could potentially be implemented for microalgae and cyanobacteria species other than those capable of growing under extreme environmental conditions. Although studies using higher volume raceway ponds are necessary, the PtxD/Phi system has the potential of opening new opportunities to exploit those strains with interesting characteristics and that are highly susceptible to contamination.

The amount and quality of microalgae/cyanobacteria seed culture are two important factors to move from the laboratory level to large-scale cultivation of microalgae/cyanobacteria as they determine the success or failure of the process [19]. During the scaling-up process, the use of variable amounts of seed inoculum have been reported in high volume bioreactors; however, in general, using seed inocula of about 10 to 25% of the final culture volume with a cell density above 1 × 10^7^ cells/mL favors a successful cultivation and avoids unwanted long lag phase and thus, might reduce the risk of contamination [15, 44]. Our results showed that using the PtxD/Phi system we were able to guarantee the quality of the seed culture throughout the different steps of the process using non-axenic conditions and reduce to 7% the inoculum required to establish a successful 100-L reactor or to 10% for a 1000-L raceway pond.

A comparative economical assessment of the Phi-based system implemented in *S. elongatus* with other control measures (i.e., antibiotics and sterilization by filtration), using raceway ponds for cultivation, suggest that the PtxD/Phi system would decrease operating costs. Using Phi as the P source and contamination control, operating costs would be 15.47 and 37% lower (Additional file 2: Tables S5 and S6) than using antibiotic [9, 50] and sterilization by filtration, respectively [51–53]. The cost of cultivation in open ponds is substantially lower than that of using other types of bioreactors, suggesting the PtxD/Phi system has great promise to contributing to overcome the contamination issue and opening the possibility of implementation of this type of bioreactor to cultivate many other microalgae/cyanobacteria species to produce biofuels and other valuable products.

In addition to phenotype stability in outdoor conditions, the ecological risk due potential accidental environmental release of genetically engineered microalgae/cyanobacteria has emerged as a major concern during large-scale cultivation [54, 55]. Evaluation in open pond production systems represents a crucial step in understanding potential ecological risk of genetically engineered cyanobacteria/microalgae. This is essential data required to develop a regulatory frame for responsible and sustainable use of genetically engineered cyanobacteria, which are key to crop improvement to meet increasing food, fuel, and value-added product demand. In the experiments performed here, potential dispersion of the genetically engineered strain was also assessed by implementing a kind of traps surrounding the raceway ponds, as reported previously [55]. It was not possible to detect the transgene in any of the tanks used as traps during the timeframe of the experiments, suggesting no dispersion of the transgenic strain under our experimental conditions. It is important to keep in mind that, whereas the PtxD/Phi system confers a competitive advantage to the engineered strain over other organisms, this trait would be only effective when Phi is present in amounts sufficient to support its growth. Although some studies have revealed a primary role of Phi on the prebiotic synthesis of phosphorylated biomolecules on early Earth, Phi have been scarcely detected in the environment [56]. Nowadays, most of the Phi present in nature might be of anthropogenic origin since it can be a byproduct of industrial processes such as metal electroplating [57] and from agricultural practices because Phi salts are used as fungicides and as growth stimulator [58]. It has been possible to detect Phi in rivers, lakes, swamps, and geothermal pools because of agricultural run-off and industrial wastewater [59–62]. In these studies, detected Phi concentrations ranged from 0.1 to 1.3 μM, which will not provide an advantage to the engineered strain over other native microalgae/cyanobacteria. Therefore, if an accidental escape occurs, also considering that high concentrations of Phi are required to sustain the normal growth (0.8 to 1.8 mM) of the transgenic strain, the Phi metabolism trait would not represent a competitive advantage in natural conditions and the transgenic strains would display similar requirements as those of the WT counterpart in a natural community. Nevertheless, it is important to consider biocontainment strategies as the ones already developed with the PtxD/Phi system to avoid any risk due accidental release to the environment and of horizontal gene transfer [21, 23]. However, as these biocontainment strategies have only been assessed on closed systems and very small scale, their validation in raceway ponds in outdoor conditions is also necessary. Our study represents the first assessment of the effectiveness and robustness of the PtxD/Phi system to control contamination during outdoor cultivation of engineered *S. elongatus*, which can be implemented for the cultivation of other microalgae/cyanobacteria.

## Conclusions

Our data show that the PtxD/Phi system has great potential for an effective control of biological contaminants in open raceway ponds for cost-effective cultivation of the cyanobacterium S*. elongatus*, which in principle should be applicable to other cyanobacteria and microalgae engineered to metabolize Phi. The expression of PtxD does not have any detectable negative effect on the fitness of the engineered strains for growth in outdoor raceway ponds and is efective as selectable marker system. Finally, since Phi is not found in nature at concentrations high enough to support the growth of engineered strains, the risk of gene dispersal or increased fitness in natural conditions is unlikely.

## Methods

### Strains, growth medium, and culture conditions

*Synechococcus elongatus* PCC 7942 (SeWT) and *Chlorella sorokiniana* UTEX 1230 were maintained and cultured using freshly prepared solid or liquid BG-11 medium (17.6 mM NaNO_3_; 0.23 mM K_2_HPO_4_; 0.3 mM MgSO_4_; 0.24 mM CaCl_2_·2H_2_O; 0.031 mM C_6_H_8_O_7_·H_2_O; 0.021 mM (NH_4_)_5_[Fe(C_6_H_4_O_7_)_2_]; 0.0027 mM Na_2_EDTA_2_H_2_O; 0.19 NaCO_2_; 1 X BG-11 trace metals solution). Solid media were prepared using 1% (w/v) Bacto-agar (Difco, Franklin Lakes, NJ) or agarose Nara Biotec^®^. Liquid cultures were maintained in an orbital shaker (MRCLOM-150DIG/500DIG) at 110 rpm. All cultures were maintained at 34 ± 1 °C and 100 μmol photons m^−2^ s^−1^ of continuous fluorescent white light.

Microalgal/cyanobacterial consortia, Comp 1 and Comp 2, were isolated from a pond water (coordinates 20°43′15.6″N 101°19′51.1″W) close to the pilot plant. During the isolation process using BG-11 [63], the collected samples were immediately subjected to the blooming process by incubation in a growth chamber at 34 ± 1 °C and irradiance of 100 μmol photons m^−2^ s^−1^ of continuous fluorescent white light and then to serial dilutions. Samples were then spread onto BG-11 agar plates and incubated for 2 weeks. Afterwards, isolated single colonies were picked up and maintained on BG-11 agar plates.

### Identification of Comp 1 based on 16S rRNA sequencing

Genomic DNA extraction was carried out as described previously [64], from cultures grown on 50 mL glass flasks with 30 mL of BG-11 media under conditions mentioned above for 4 days. Genomic DNA was analyzed by Macrogen Inc (South Korea) for amplification of 16S rRNA gene using the primers 785F (5′-GGATTAGATACCCTGGTA-3′), 907R (5′-CCGTCAATTCMTTTRAGTTT-3′), 27F (5′-AGAGTTTGATCMTGGCTCAG-3′), and 1492R (5′-TACGGYTACCTTGTTACGACTT-3′). PCR products were sequenced by Sanger [65], using an ABI Prism 3730XL DNA analyzer (Applied Biosystems, Macrogen Inc, Seoul, South Korea). The sequences obtained were analyzed using Basic Local Alignment Search Tool (BLASTN) and matched against non-redundant nucleotide collection (nr/nt) present in NCBI GenBank database (http://www.ncbi.nlm.nih.gov).

### Plasmid construction and genetic transformation

The *ptxD* encoding gene from *P. stutzeri* WM88 (AF061070, http://www.ncbi.nlm.nih.gov/nuccore/AF061070) was codon-optimized to the nuclear codon usage of *S. elongatus* according to the OptimumGene™-Codon optimization Tool (GenScript, Piscataway, NJ) and placed under control of the *psbAI* constitutive promoter of the psyn_6 vector (Life Technologies Corporation, Carlsbad, CA) [27]. psyn_6 harbors a spectinomycin resistance cassette for the selection of *E. coli* and *S. elongatus* and has NS1 (neutral site 1) homologous recombination sites for the integration of the TDNA into the *S. elongatus* genome. NS1a and NS1b sites are also present on *S. elongatus* genome to guide double homologous recombination of DNA contained between the neutral sites in the vector [66]. The *ptxD* gene was synthesized with the required restriction sites and cloned into *Nde*I and *Nsi*I sites to create the plasmid psyn_6_PtxDopt. The resulting plasmid was then subjected to restriction digestion analysis using *EcoR*I and *Bgl*II and sequencing analysis to corroborate the correct *psbAI*::*ptxD* gene construct.

The wild type strain *S. elongatus* PCC 7942 used in this study was then transformed via natural for genomic integration of exogenous genes according to the user’s guide of GeneArt ^®^*Synechococcus* Protein Expression Vector (Life Technologies Corporation). Briefly, 15 mL of culture from middle logarithmic phase at 8.7 × 10^8^ cells/mL were collected by centrifugation and resuspended in 300 μL BG-11 without any source of P in the medium. Plasmid DNA (200 ng) was added to the cell suspension and incubated in dark for 16 h at 34 °C and 110 rpm. Transformants were selected using BG-11 agar plates supplemented with 100 μg/mL spectinomycin as a selective agent. Engineered strains were then tested for their capacity to grow on Phi medium as the sole P source using multiwell culture plates and/or 50 mL glass flasks. Selected transgenic strains were always maintained in Phi containing medium for future experimentation.

### PCR colony analysis

Colonies grown onto BG-11 agar plates under conditions mentioned above were picked out and resuspended in 5–10 μL DMSO and subjected to heat at 95 °C for 5 min. One microlitre of the resultant suspension was used as a template for PCR. SeptxD primers, Fw (5′-CTCTGCTGGTCAATCCGTGT-3′) and Rv (5′-GCCTTGGGCAGGCGATTA-3´) were used to amplify a 304 base pairs (bp) fragment of the *ptxD* gene using Taq DNA Polymerase Recombinant (Life Technologies). The thermocycler was programmed as follows: 94 °C for 3 min, denaturation (94 °C for 30 s), annealing (64 °C for 30 s) and extension (72 °C 1 m) for 34 cycles, then a final extension at 72 °C for 7 min. The PCR products were examined on 1% agarose gel using SYBR-Safe DNA gel stain (Life Technologies).

### Insertion of the expression cassette at NS1

Strains SeptxD-1 to -6 grown onto BG-11 agar plates supplemented with Phi were picked out and resuspended in 5–10 μL DMSO and subjected to heat at 95 °C for 5 min. One microlitre of the suspension was used as a template for the PCR. Three different combinations of primer were utilized to verify the correct insertion: (1) NS1 primers, Fw (5′-CTACCGAAGTCGCTCGTAG-3′) and Rv (5′-CTATGGTTCGGGATCACTG-3′), (2) NS1_Fw with SeptxD_Rv, and (3) Ns1_Rv with SeptxD_Fw, which resulted in fragments of 3288, 2739, and 804 bp, respectively. The thermocycler was programmed as follows: 98 °C for 3 min, denaturation (98 °C for 30 s), annealing (60 °C for 30 s) and extension (72 °C 1 m) for 34 cycles, then a final extension at 72 °C for 7 min, with Phusion^®^ High-Fidelity DNA Polymerase (New England Biolabs). After examination of the PCR products on 1% agarose gel using SYBR-Safe DNA gel stain (Life Technologies), the expected fragments were cut-off from the gel, purified using Zymoclean Gel DNA Recovery Kit (Zymo Research), and subjected to Sanger DNA sequencing [65] by Macrogen Inc, Seoul, South Korea. Sequences were analyzed with Genious 2019.0.4 (https://www.geneious.com) and trimmed to eliminate low quality bases.

### Real-time quantitative PCR

Total RNA was isolated using the PureZOL™ RNA isolation Reagent (Bio-Rad, Hercules, CA) following the manufacturer’s instructions. The purified total RNA concentration was determined using a NanoDrop 2000 spectrophotometer (Thermo Scientific, Waltham, MA), and its quality and integrity were corroborated by agarose gel electrophoresis (1.2%). Subsequently, 10 μg of RNA was treated with *DNase*I (New England Biolabs, Ipswich, MA) to eliminate any contaminating genomic DNA. Gene-specific primer to amplify *ptxD* (SeptxD primers), *SerpoD* (Fw: 5′-AGATGGTGCAGTCGAACCTG-3, Rv: 5′-GAGTGGCGATCTCTTCCTCG-3′), and *SernpB* (Fw: 5′-AAAAGACCAGACTTGCTGGGT-3, Rv: 5′-CGAAGACAGAGGGCAGTTATC-3′) genes were designed using the NCBI/Primer-BLAST tool [67]. *SerpoDA* and *SernpB* were used as housekeeping genes for cDNA content normalization [68]. cDNAs were synthesized by adding 2 μM of gene-specific reverse primers and 500 μM of dNTPs mix to 500 ng of total RNA. This mixture was incubated at 65 °C for 5 min and briefly chilled on ice. First Strand Buffer (Invitrogen), 20 mM of dithiothreitol (DTT) and 200 units of Superscript III (Invitrogen) were added to the prior mixture to a total volume of 20 μL and incubated at 55 °C for 1 h following manufacturer’s instructions. Inactivation of the reverse transcriptase was done by incubating the mixture at 70 °C for 15 min and the cDNA solution was stored at − 20 °C. PCR (Polymerase Chain Reaction) was performed using the SensiFAST SYBR No-ROX Kit (BIOLINE) in MIC qPCR Magnetic Induction Cycler (BIOLINE) system. Reaction mixtures contained 1 μL cDNA, 400 nM of each primer and SensiFAST SYBR^®^ No-ROX Mix, in a total volume of 10 μL. Reaction mixtures were incubated for 2 min at 95 °C, followed by 40 amplification cycles of 5 s at 94 °C, 10 s at 60 °C and 20 s at 72 °C. Results were analyzed using the MIC qPCR Cycler on-board software (BIOLINE). The qPCR was conducted with at least three experimental replicates for each biological sample.

### PtxD activity determination

The enzymatic activity of PtxD was measured using total protein extracts of *S. elongatus* cells. 0.2 g of freshly collected wet biomass, obtained by centrifugation and removing the supernatant with a pipette, were weighed, washed twice with 1 mL of 50% acetone to extract the chlorophyll, and then centrifuged at 4000 rpm for 5 min at 4 °C. The pellet was resuspended in 1 mL of lysis buffer (50 mM MOPS pH 7.25, 150 mM NaCl, 5% glycerol, 5 mM β-mercaptoethanol, 1 mM PMSF) and sonicated with six short pulses bursts of 30 s followed by intervals of 30 s for cooling on ice, at an amplitude of 40%. To lower cell debris, the samples were centrifuged at 20,000 rpm for 30 min at 4° C. Total protein concentration of the supernatant was determined by Bradford method (Bradford, 1976) using the Quick Start™ Bradford dye (BioRad), following the supplier’s specifications. PtxD activity was determined by measuring the fluorescence emitted by NADH. The reaction mixture was prepared at a final concentration of 50 mM MOPS (pH 7.25), 0.5 mM NAD^+^, 1 mM phosphite, and 50 μg total protein (protein extract), in a final volume of 250 μL. The fluorescence intensity was measured after 1 h of incubation at 30 °C in a fluorescence reader (Fluoroskan Ascent™ Microplate Fluorometer), at an excitation and emission wavelength of 340 and 460 nm, respectively. Linear increase of the fluorescence using protein extract of the transgenic strain was verified under these conditions (Additional file 1: Figure S16).

### Growth of *S. elongatus* PCC 7942 and transgenic strains on phosphite media

For the experiments to study the capacity of *S. elongatus* PCC7942 (SeWT) and the transgenic strains to use Phi, the source of P on the conventional BG-11 media, potassium phosphate dibasic (K_2_HPO_4_, 0.2 mM) was substituted by potassium phosphite monobasic (KH_2_PO3, Wanjie International CAS No. 13977-65-6) at different concentrations as noted for each experiment, using standard BG-11 and BG-11 devoid of P media as controls. SeWT was cultivated in 50 mL glass flask with 30 mL media, inoculated at 1% (v/v), and incubated at 34 °C, 110 rpm and 100 μmol photons m^−2^ s^−1^ of continuous fluorescent white light. SeWT inoculum was produced using Pi-containing media (0.2 mM). Growth was estimated by measuring cell density using a Neubauer chamber. All the experiments were conducted in triplicate during a period of 8 days after inoculation.

### PtxD/phosphite system as a selectable marker

To determine the tolerance of SeWT to spectinomycin and Phi, 80 µL of a P-starved culture at 8.7 × 10^8^ cells/mL in middle logarithmic phase, were inoculated in BG-11 media with 0, 5, 10, 20, 30, 50 and 100 µg/mL spectinomycin or 0.1, 0.2, 0.5, 1.8, and 2 mM Phi in multiwell plates or agar plates. For the selection of transgenics, three different amounts (1.7, 6.1, and 8.7 × 10^8^) of cells previously recombined as described above with the expression cassette *psbAI*::*ptxDopt,* were directly spread onto agar plates with 100 μg/mL spectinomycin or 0.5 and 1.8 mM Phi. Isolated colonies were counted manually after 15 days.

### Growth competition assays

*Chlorella sorokiniana* UTEX 1230 (CsWT) and the two microalgal/cyanobacterial consortium (Comp 1 and Comp 2) were used as the competitors for in vitro assays. For the competition experiment with CsWT, proportions 1:1, 1:4, and 4:1 of inocula *C. sorokiniana*:SeptxD-2 were used, whereas for experiments with Comp 1 and Comp 2 proportion 1:1 (SeptxD-2:Comp) was implemented. The competition cultures were grown in BG-11 media supplemented with 1.8 mM Phi or 0.2 mM Pi as the phosphorus (P) source. Monocultures of both the microalga and the cyanobacterium were used as controls. Experiments were carried out using 50 mL glass flasks with 30 mL of media, under culture conditions described above. Each treatment was performed in triplicate. The growth was estimated by cell counting with a Neubauer chamber. For experiments under non-sterile conditions, sterilization of materials and growth media were avoided to allow microorganisms to invade the cultures. Cultures were observed and photographed using a Zeiss Axio Lab.A1 microscope.

### Scale-up of liquid cultures, outdoor cultivation and biosafety regulation

#### Scale-up of liquid cultures for inocula preparation

For inocula preparation, 25 mL cultures of the strains were started by scraping a portion of the culture from a plate and re-suspending in 5 mL of media before added to the 50 mL flask. 20 mL cultures were then passed directly to 700 mL cultures in 1  L glass bottles or Erlenmeyer flasks and then to 7  L of media in 10-L home-designed cylindrical bioreactors. 20 mL cultures were grown in an orbital shaker at 110 rpm, 34 ± 1 °C, 100 μmol photons m^−2^ s^−1^ of continuous fluorescent white light, whereas 700 mL cultures were bubbled with air at a flow rate of 2 L/min. Autoclaved BG-11 media were used for cultivation in Petri dishes, in 50 mL and 1-L containers, whereas non-sterile media were used for the rest of the process. pH was adjusted to 7 in all freshly prepared media. For experiments under non-sterile conditions, sterilization of materials and growth media were avoided to allow microorganisms to invade the cultures.

#### Cultivation in 10-L cylindrical bioreactors

After growth, 700 mL cultures were used to inoculate 7  L of media in 10-L home-made cylindrical bioreactors and incubated at 100 μmol photons m^−2^ s^−1^ of continuous fluorescent white light, and using charcoal-filtered dechlorinated municipal tap water. Cylindrical bioreactors of 45 cm height, 20 cm internal diameter, and 7 L working volume, were constructed with acrylic using a 12 mm thick sheet PLASTICRYL purchased from Brunssen de Occidente (item #0171-0010-012) from Guadalajara, Jalisco, Mexico. Air was supplied and maintained to each culture at a flow rate of approximately 10 L/min by manually adjusting tubing connections to a small pump. We did not detect any observable growth variations for any culture in reference to air flow-rate.

Cell counting and pH was monitored every day during the timeframe of the experiments. As we detected no significant pH variations throughout the experiments, pH was not subsequently adjusted. Samples were withdrawn through the vent holes in the cap using glass pipettes. During the continuous cultivation, only 6.5 mL were removed and the bioreactors refilled with BG-11 media. To avoid cross contamination between the transgenic strains, tubing and accessories were washed using 1.5 mg/L calcium hypochlorite and flushed with water.

#### Cultivation in 100-L cylindrical bioreactors

Outdoor experiments were performed in StelaGenomics México facility located in Irapuato Guanajuato Mexico (20°42′56.2″N 101°20′16.4″W). Cylindrical bioreactors of 1.45 m height, 35 cm internal diameter, and 100-L working volume (110 L total volume), were constructed with acrylic using a 12 mm thick sheet purchased from Brunssen de Occidente from Guadalajara, Jalisco. Each cylindrical bioreactor was installed in a robust metal structure and suspended about 1.95 m above soil level. The air was supplied and maintained to each column at a flow rate of approximately 20 L/min by manually adjusting a blower; an internal removable stainless-steel diffuser device was installed in each column. Separate PVC lines were installed in each cylindrical bioreactor to allow culture transfer to 1000-L raceway ponds. CFU from contaminating bacteria was estimated by serial dilutions (1:10, 1:100, and 1:1000) of the sample and plating onto Petri dishes with LB (Luria–Bertani) medium. The plates were then incubated at 37 °C for 48 h to favor bacterial growth. As controls, SeptxD-2 strain was also plated onto LB and BG-11 agar plates and incubated under the same conditions. CFU was manually counted and data analyzed.

#### Cultivation in 1000-L raceway ponds

Following growth in cylindrical bioreactors, the culture was used to inoculate 1000-L raceway ponds. For the raceway ponds, we constructed a carbon steel structure with an oval shape (1.5 m width, 3.5 m in length, 60 cm depth), which were covered with a HDPE (high-density polyethylene) geomembrane. Raceways were installed 25 cm above soil level and operated at a depth of 25 cm. A stainless-steel propeller device was installed to recirculate the culture, which was operated at about 10 cm depth and rotated at the speed of 15 rpm.

Optical density (OD_750_), pH, temperature of the media, and solar irradiance were recorded every day at 9 am and 6 pm during the timeframe of the experiments. As we detected no significant pH variations throughout the experiments, pH was not subsequently adjusted. Biomass concentration was determined after 7 days of cultivation.

For outdoor experiments, non-sterile BG-11 media were prepared using industrial grade reagents: NaNO_3_ (ISAAQUIM, ERI52LO0619), MgSO_4_·7H2O (ISAAQUIM, S114773046), CaCl2·2H_2_O (ISAAQUIM, CC51110120), KHPO_4_ (ISAAQUIM, 011771924431), (HOOCCH_2_)_2_C(OH)COOH, (NH_4_)_5_[Fe(C_6_H_4_O_7_)_2_] (ISAAQUIM, 82239488QO), Na_2_CO_3_ (ISAAQUIM, 090120).

Environmental conditions during the timeframe of the experiments can be seen directly at https://weatherspark.com/y/4553/Average-Weather-in-Irapuato-Mexico-Year-Round.

#### Biosafety regulation

Based on the provisions of Mexico’s Biosafety Law of Genetically Modified Organisms, on August 15, 2014, StelaGenomics submitted a notice for the Confined Use of Genetically Modified Organisms (application no. 09/J7-0081/08/14) to the Secretariat of Environment of Mexico (SEMARNAT), whereby the company stated that the activities with genetically modified organisms would be the culture of microalgae, cyanobacteria, and other microorganisms, in cylindrical reactors and open raceways ponds located in the facilities of StelaGenomics, applying the appropriate biosafety measures. On March 22, 2016, SEMARNAT delivered a favorable opinion, through the document SGPA/DGIRA/DG/1639, in which the aforementioned activities were approved, as long as the biosafety measures stated in the notice are applied. One of the *S. elongatus* strains generated under this application, SeptxD-2, was selected for outdoor cultivation. Some of the biosafety measures implemented are: access to the facility is restricted by a perimeter fence and there is personal and video surveillance 24 h/day; bioreactors and raceways ponds are installed on a cement platform covered with an industrial polymer (or with a high-density polyethylene impermeable geomembrane) to retain and control any potential leak and to avoid leaks to the soil; the core facility is surrounded by a 30 cm perimeter barrier to prevent the escape of biological material to the environment in case of accidental spill caused by flooding or excessive rain; this platform has PVC connections to discharge potential spilled liquids into a 10,000-L plastic container (Rotoplast) installed belowground, where the liquids are chlorinated and treated with an UV lamp, before recycling into the process; 7-L cylindrical reactors are covered with acrylic caps; raceways ponds are covered with an anti-bird netting and anti-aphid mesh, preventing the access of any type of birds and insects that could spread microalgae/cyanobacteria in the surrounding area; after harvesting to determine production of biomass, waste water is chlorinated, UV treated, and recirculated into the system.

### Dispersal experiment

To examine the potential dispersion of the transgenic strain from the raceway ponds to the environment, we implemented a kind of traps surrounding the pilot plant. Traps consisted of 1200-L plastic containers placed on the north, south, and east directions from the source cultivated pond, filled with 500  L water and supplemented with BG-11 medium (Additional file 1: Figure S13). In east direction, three traps were placed at 3, 6, and 28 m from the raceway facility. In the north and south, only one tank was placed at 1.5 m from the raceway. 50 mL samples were collected from the containment tanks three times per week and preserved at − 20 °C for PCR and RT-qPCR analysis. One mL of each sample was then taken for analysis. Upon centrifugation at 4200 rpm for 20 min, the pellet was resuspended in 0.5 mL DMSO and subjected to heat at 95 °C for 5 min. One or two microlitre of the suspension were used as a template for the PCR and RT-PCR analysis using SeptxD primers as described above.

### Statistical analysis

Statistical analysis was performed using the program R 3.5.2. Data collected from the different experiments were subjected to paired Student’s *t* test, with Bonferroni correction or one sample Student’s *t* test. *P* values < 0.05 were considered significant (*P < 0.05, **P < 0.01, ***P < 0.0001). One-way ANOVA and Tukey’s multiple comparison test were applied to analyze gene expression.

## Supplementary information

**Additional file 1.** Additional figures and legends supporting the results described in the text.

**Additional file 2.** Additional tables and legends supporting the results described in the text.

## Data Availability

Data sharing is not applicable to this article as no datasets were generated or analyzed during the current study.

## Abbreviations

PtxD: Phosphite oxidoreductase
Phi: Phosphite
Pi: Phosphate
P: Phosphorus
SeptxD: *S. elongatus* PCC 7942 transgenic strains transformed with a codon-optimized version of *ptxD* gene
SeW: *S. elongatus* PCC 7942 wild type
NS1: Neutral site 1
CsWT: *Chlorella sorokiniana* UTEX 1230 wild type
Comp 1: Competitor 1
Comp 2: Competitor 2
CFU: Colony forming units
LB: Luria–Bertani
NAAB: National Alliance for Advanced Biofuels and Bioproducts
ASP: Aquatic Species Program
HDPE: High-density polyethylene
