# Supplementary material for: Metabolic engineering of phosphite metabolism in Synechococcus elongatus PCC 7942 as an effective measure to control biological contaminants in outdoor raceway ponds
Source: Biotechnol Biofuels. 2020 Jul 9;13:119. doi: 10.1186/s13068-020-01759-z (PMC7346359; doi:10.1186/s13068-020-01759-z)
Supplement: Supplementary file 1 — Additional file 1. Additional figures and legends supporting the results described in the text. [file 13068_2020_1759_MOESM1_ESM.pdf]

Additional file 1

Metabolic engineering of phosphite metabolism in *Synechococcus elongatus* PCC 7942 as an effective measure to control biological contaminants in outdoor raceway ponds

Sandra Isabel González-Morales[1], Navid Pacheco-Gutiérrez[1], Carlos A. Ramírez-Rodríguez[1], Alethia A. Brito-Bello[1], Priscila Estrella-Hernández[1], Luis Herrera-Estrella[2,3], Damar L. López-Arredondo[1,3,§]

[1]StelaGenomics México, S de RL de CV, Av. Camino Real de Guanajuato s/n, 36821 Irapuato, Guanajuato, Mexico.

[2]Laboratorio Nacional de Genómica para la Biodiversidad, Unidad de Genómica Avanzada del Centro de Investigación y de Estudios Avanzados del Instituto Politécnico Nacional, Km 9.6 carretera Irapuato León, Irapuato, 36500 Guanajuato, Mexico.

[3]Institute of Genomics for Crop Abiotic Stress Tolerance, Texas Tech University, Lubbock, TX, 79409 USA.

[§]To whom correspondence should be addressed:

Damar López-Arredondo, email: Damar.Lopez-Arredondo@ttu.edu; Tel. 806 8343364, orcid: https://orcid.org/0000-0001-7389-3143

a

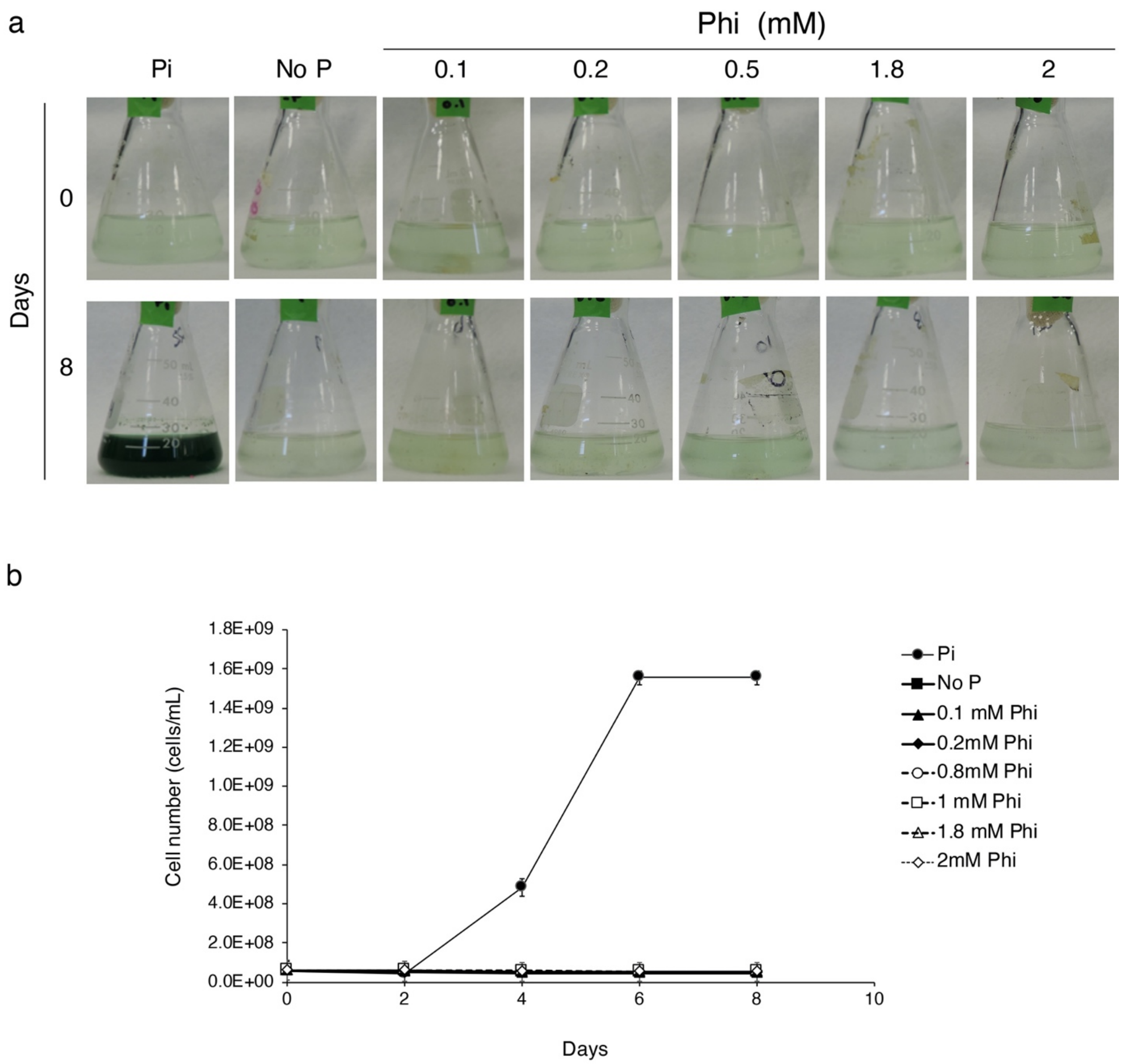

Figure S1. Effect of phosphite on *Synechococcus elongatus* PCC 7942 growth. a) *S. elongatus* PCC 7942 (SeWT) was grown in BG-11 media supplemented with 0.1, 0.2, 0.5, 1.8, and 2 mM phosphite (Phi), and in media with phosphate (Pi, 0.2 mM) and without a phosphorus (P) source (No P) as controls. Photographs show cultures at day cero and eight after inoculation. b) Cell number (cells/mL) was determined every two days during the timeframe of the experiment. Data shown are the average of three replicates ± SD.

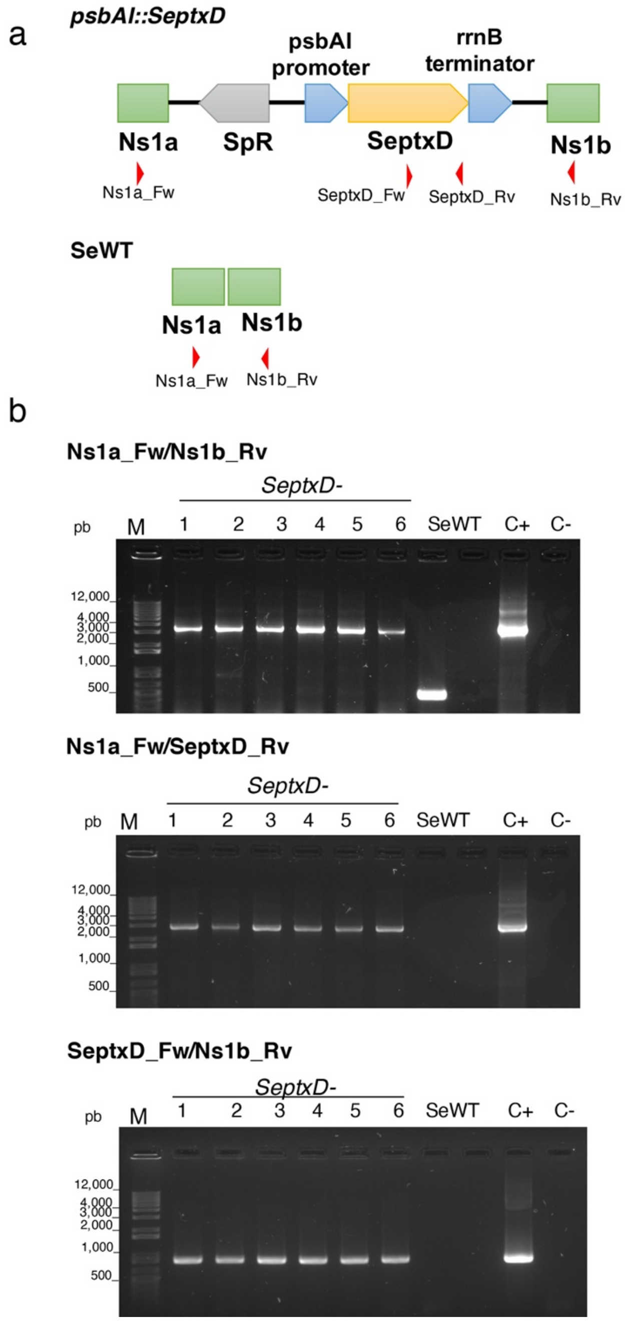

Figure S2. Insertion of the *psAbI::SeptxD* expression cassette at NS1. a) Schematic representation (top) of the genomic organization at neutral site 1 (NS1). The cassettes, including spectinomycin resistance (SpR) and SeptxD gene, were integrated at NS1 by homologous recombination. b) Complete segregation of the transformants was confirmed by PCR with the appropriate primer pairs represented by red arrowheads in the upper panel (a). The target sizes of each PCR product were 3,288 pb for NS1a_Fw/NS1b_Rv, 2,752 pb for NS1a_Fw/SeptxD_Rv, and 840 pb for SeptxD_Fw/NS1b_Rv. SeWT: wild type; 1kb: 1kb plus ladder (Invitrogen); C(+): positive control, plasmid; C(-): negative control, water.

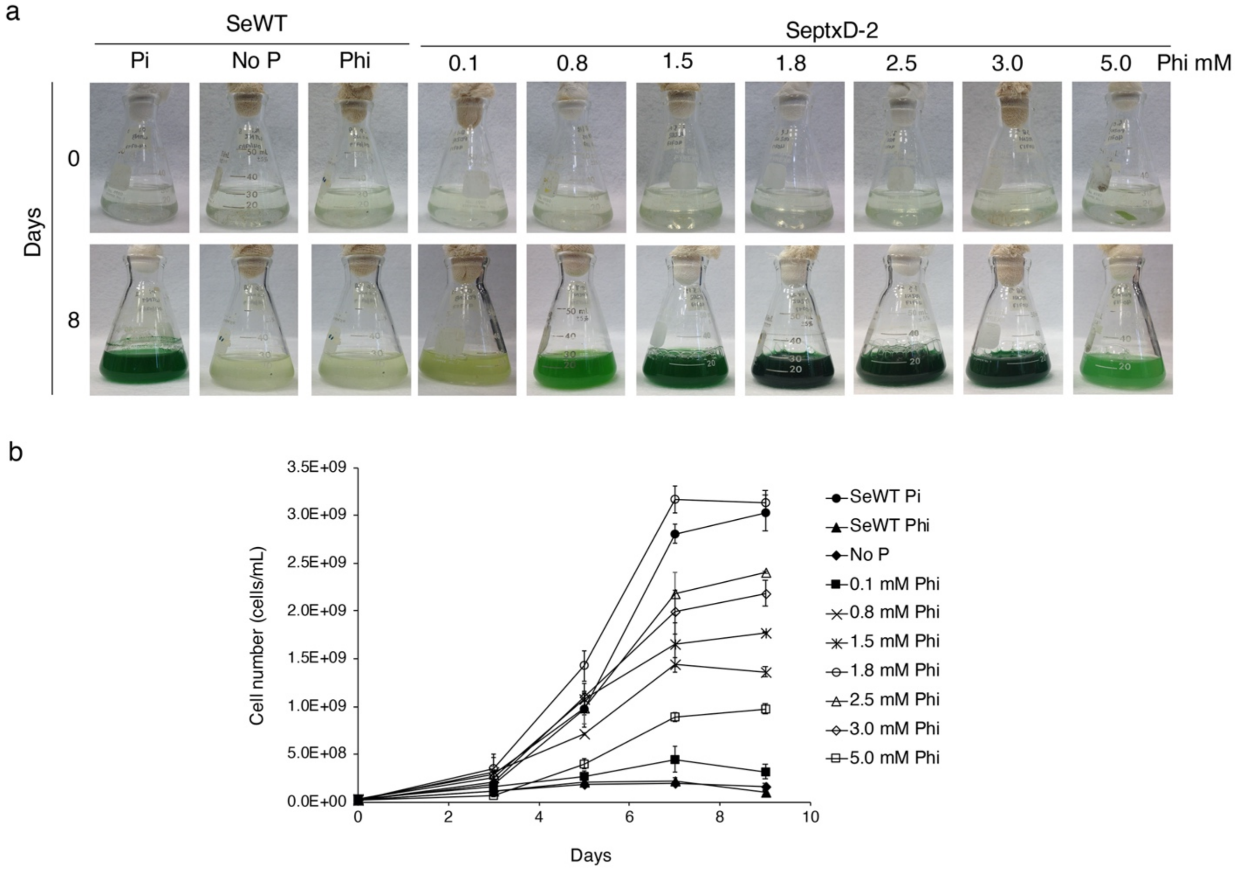

Figure S3. Growth assessment of SeptxD-2 transgenic strain in different concentrations of phosphite. a) Growth of *S. elongatu*s transgenic strain SepxtD-2 was assessed in BG-11 media supplemented with 0.1, 0.8, 1.5, 1.8, 2.5, 3, and 5 mM phosphite (Phi). Media with phosphate (Pi, 0.2 mM), with Phi (1.8 mM), and without a phosphorus (P) source were used as controls to grow *S. elongatus* PCC 7942 (SeWT). b) Cell number (cells/mL) was determined every two days during the timeframe of the experiment. Data shown are the average of three replicates ± SD.

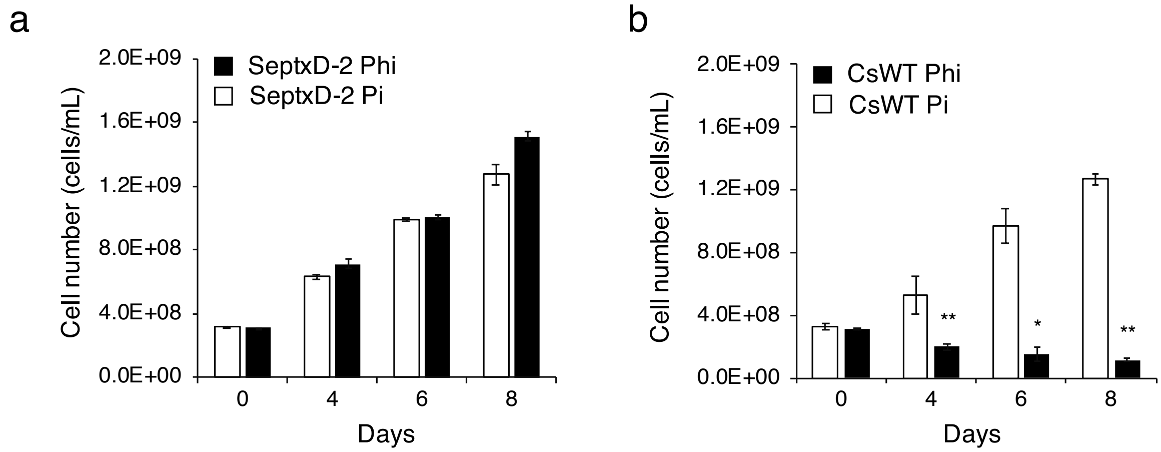

Figure S4. Monocultures of SeptxD-2 and *C. sorokiniana* in competition experiments. Cell number (cells/mL) of SeptxD-2 (a) and *Chlorella sorokininana* (CsWT) (b) cultured in phosphate (Pi) and phosphite (Phi) media, was determined four, six, and eight days after inoculation when grown as monoculture as part of the competition experiments in Figure 2. Data shown are the average of three replicates ± SD (Student's t-test, *$P < 0.05$, **$P < 0.01$, ***$P < 0.0001$).

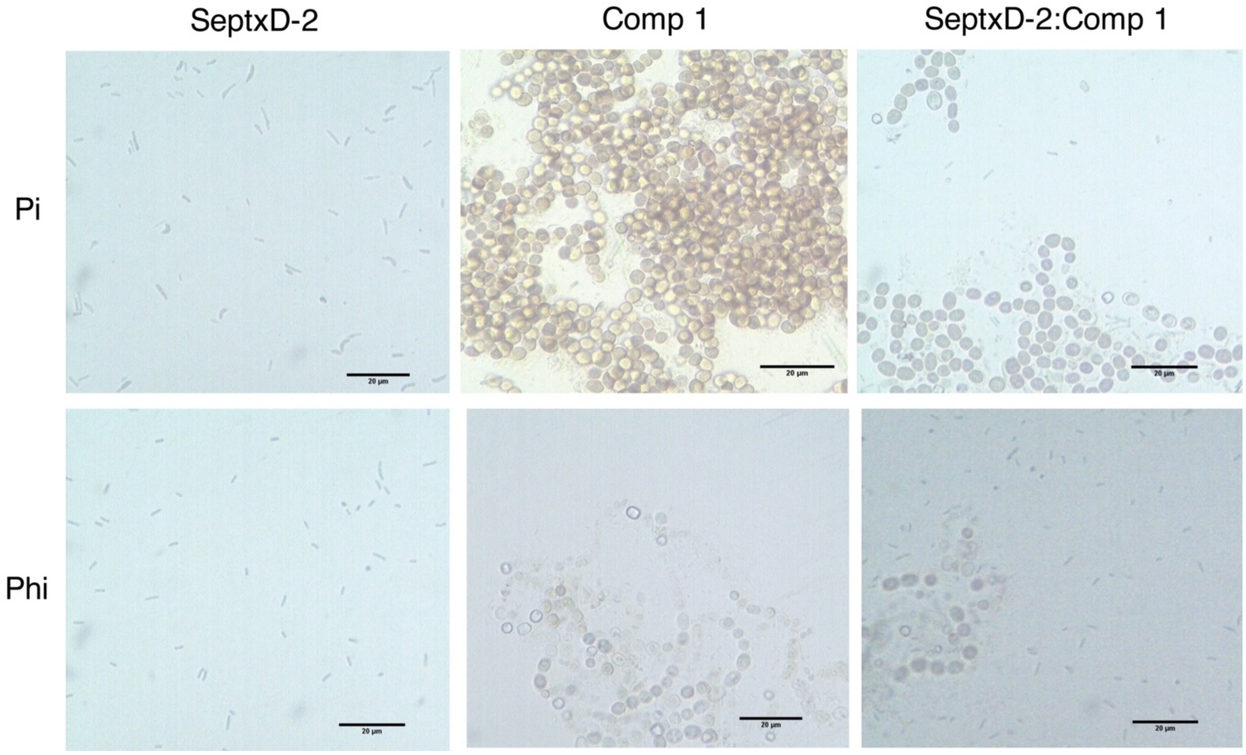

Figure S5. Photographs of SeptxD-2 and the natural Competitor 1 during competition experiments. Light microscopy images of the competition experiments between SeptxD-2 and a natural competitor (Comp 1), as monocultures and mixed cultures (1:1, SeptxD-2:Comp 1) in media with phosphate (Pi) and Phosphite (Phi) as phosphorus source. Scale bar corresponds to 20 µm.

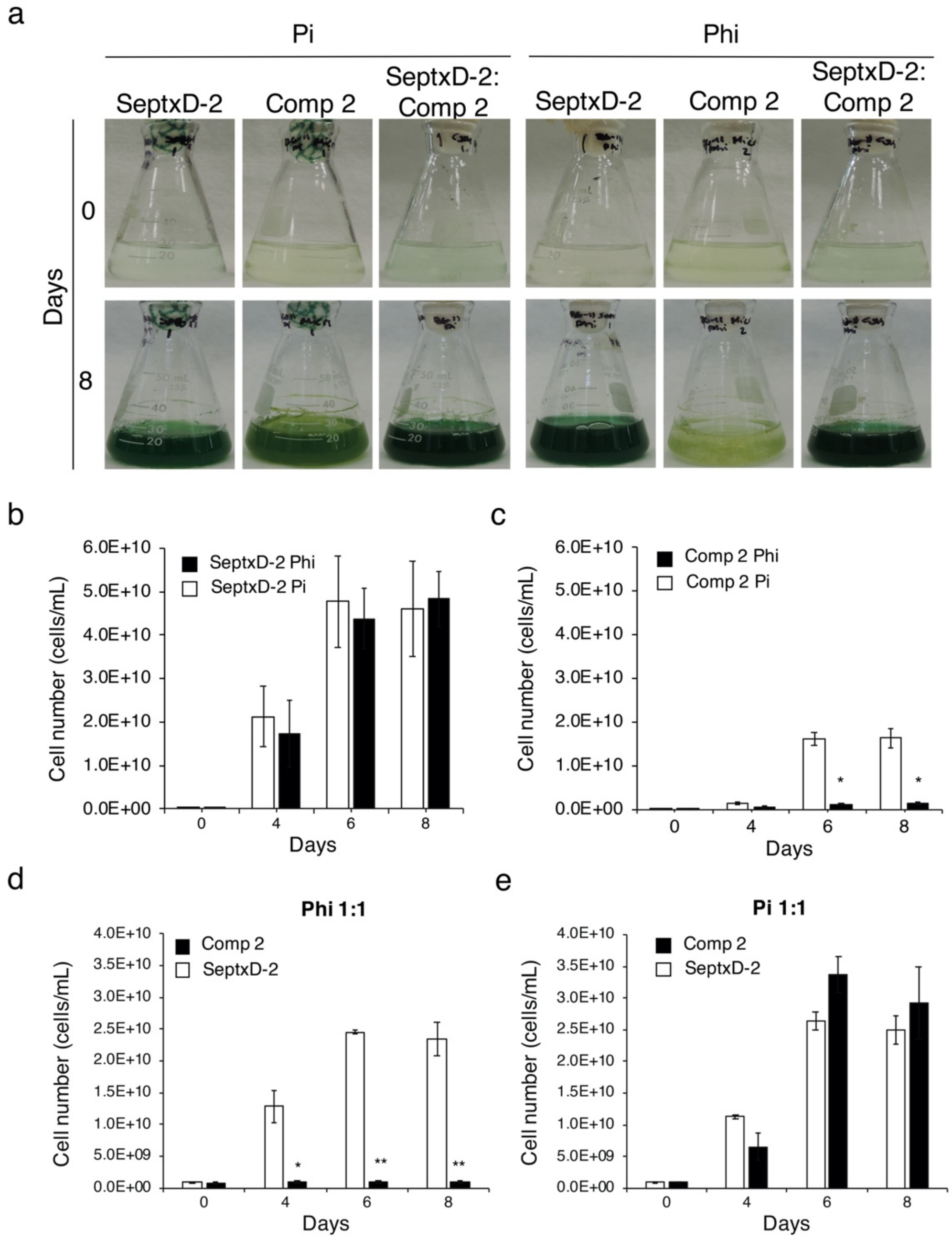

Figure S6. Growth competition experiment between SeptxD-2 and the natural Competitor 2. a) *S. elongatus* transgenic strain (SeptxD-2) and natural competitor (Comp 2) were grown as monocultures and mixed cultures using BG-11 medium supplement with 0.2 mM phosphate (Pi) or 1.8 mM phosphite (Phi) as phosphorus source. Cultures were photographed eight days after the inoculation. Cell number (cells/mL) of the monocultures of SeptxD-2 (b) and the natural competitor 2 (Comp 2) (c), and for the mixed cultures under Phi (d) and Pi (e) treatments, was determined. Values are the mean of three replicates ± SD. Bars with asterisks are significantly different from the control (Student's t-test, $*P < 0.05$, $**P < 0.01$, $***P < 0.0001$).

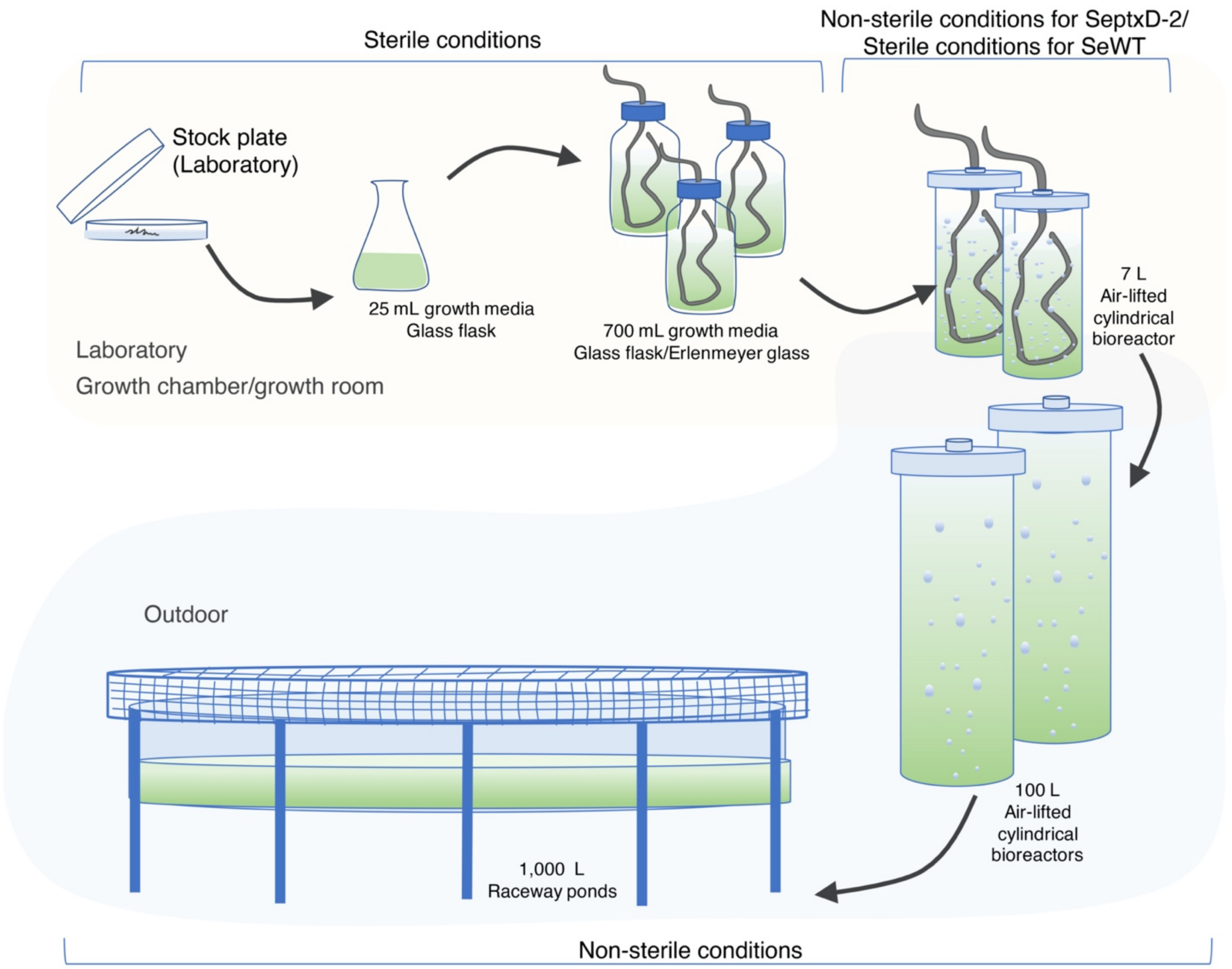

Figure S7. Illustration of the scaled-up cultivation of *S. elongatus*. Under laboratory conditions, 25 mL cultures of the strains were started by scraping a portion of the culture from a plate. After 8 days of culture, 20 mL of the culture were used to inoculate 700 mL of growth media in 1 L glass bottles. 600 mL of this culture were passed to 7 L home-designed cylindrical bioreactors. These cultures were then used to inoculate 100 L cylindrical reactors outdoor, and these to inoculate 1,000 L raceway ponds. Raceways ponds were covered with an anti-bird netting and anti-aphid mesh to prevent the access of any type of bird and insect that could spread microalgae in the surrounding area.

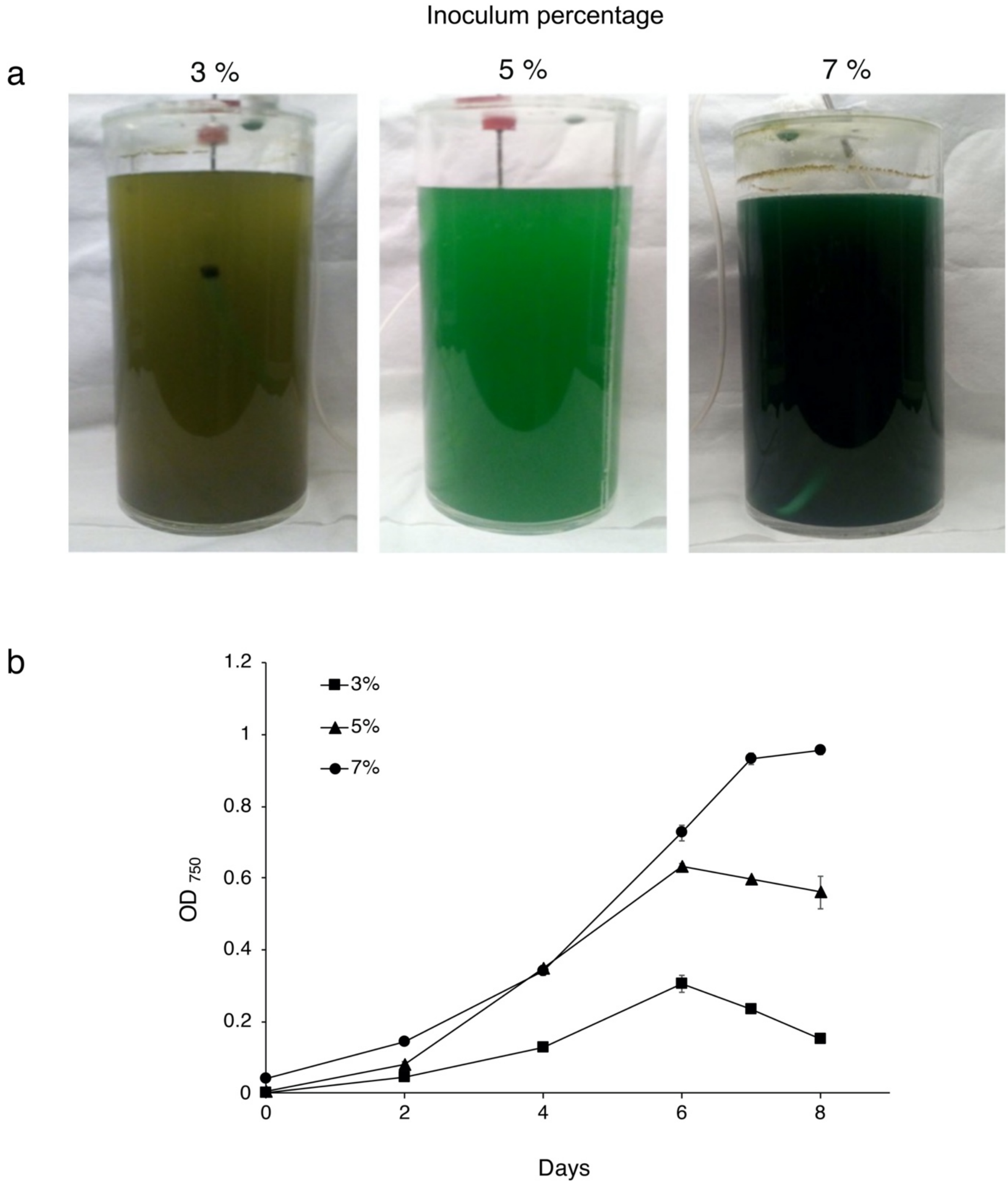

Figure S8. Inoculum percentage optimization for SeptxD-2 cultivation in 7 L cylindrical reactors. a) *S. elongatus* transgenic strain (SeptxD-2) was grown in BG-11 media prepared with industrial grade reagents and supplemented with 1.8 mM of phosphite (Phi) as P source. Cultures were performed using 3, 5, and 7% (v/v) inoculum in non-sterile conditions. b) Optical density (OD_{750}) of the cultures grown under controlled conditions and bubble with air, was determined every two days. Data shown are the average of three replicates ± SD.

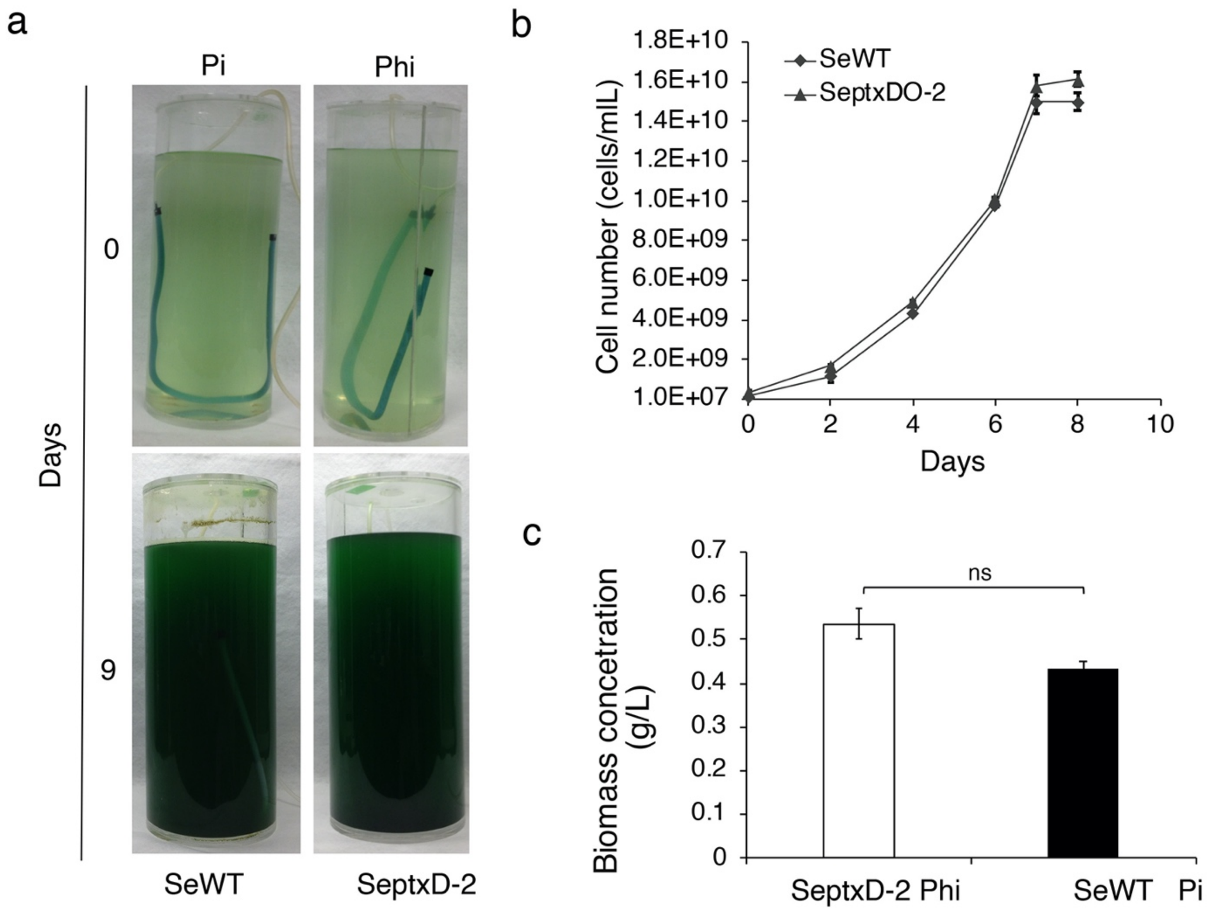

Figure S9. Growth of SeptxD-2 in 7 L cylindrical photoreactor using phosphite as phosphorus source. (a) *S. elongatu*s transgenic strain (SeptxD-2) under non-axenic conditions and wild type *S. elongatus* PCC 7942 (SeWT) under axenic conditions, were grown in BG-11 media prepared with industrial grade reagents and supplemented with 0.2 mM phosphate (Pi) or 1.8 mM phosphite (Phi) as P source. Cultures were performed using 7 % (v/v) inoculum in 7-L cylindrical bioreactors. b) Cell number (cells/mL) of cultures on (a) was determined every two days during eight days. (c) Accumulated dry biomass (g/L) after the cultivation period. Values are the means ± SD of three replicates. Bars with asterisks are significantly different from the control (Student's t-test, *P < 0.05, **P < 0.01, ***P < 0.0001, ns: no significant differences).

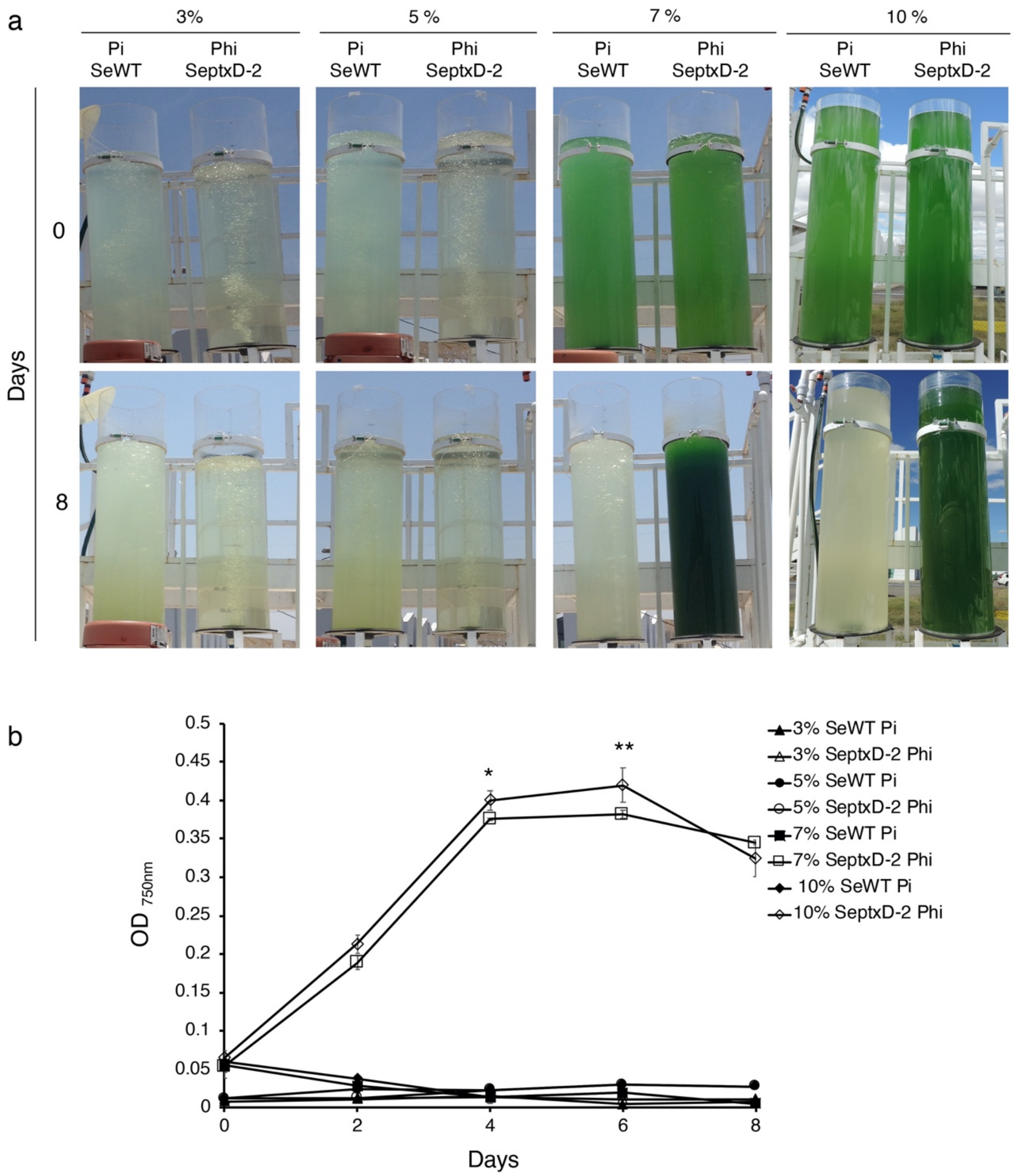

Figure S10. Growth of SeptxD-2 in 100 L cylindrical photoreactor with different inoculum percentage using phosphite as phosphorus source under non-axenic outdoor conditions. (a) *S. elongatus* transgenic strain (SeptxD-2) and wild type *S. elongatus* PCC 7942 (SeWT) were grown in BG-11 media prepared with industrial grade reagents and supplemented with 0.2 mM of phosphate (Pi), or 1.8 mM of phosphite (Phi) as P source. Cultures were performed using 3, 5, 7, and 10 % (v/v) inoculum, in non-sterile 100 L cylindrical bioreactor in outdoor conditions. b) Optical density (OD_{750}) was determined every two days for eight days. Data shown are the average of three replicates ± SD. Points with asterisks are significantly different with respect to the culture inoculated with 7% (v/v) (Student's t-test, *$P < 0.05$, **$P < 0.01$, ***$P < 0.0001$).

a

b

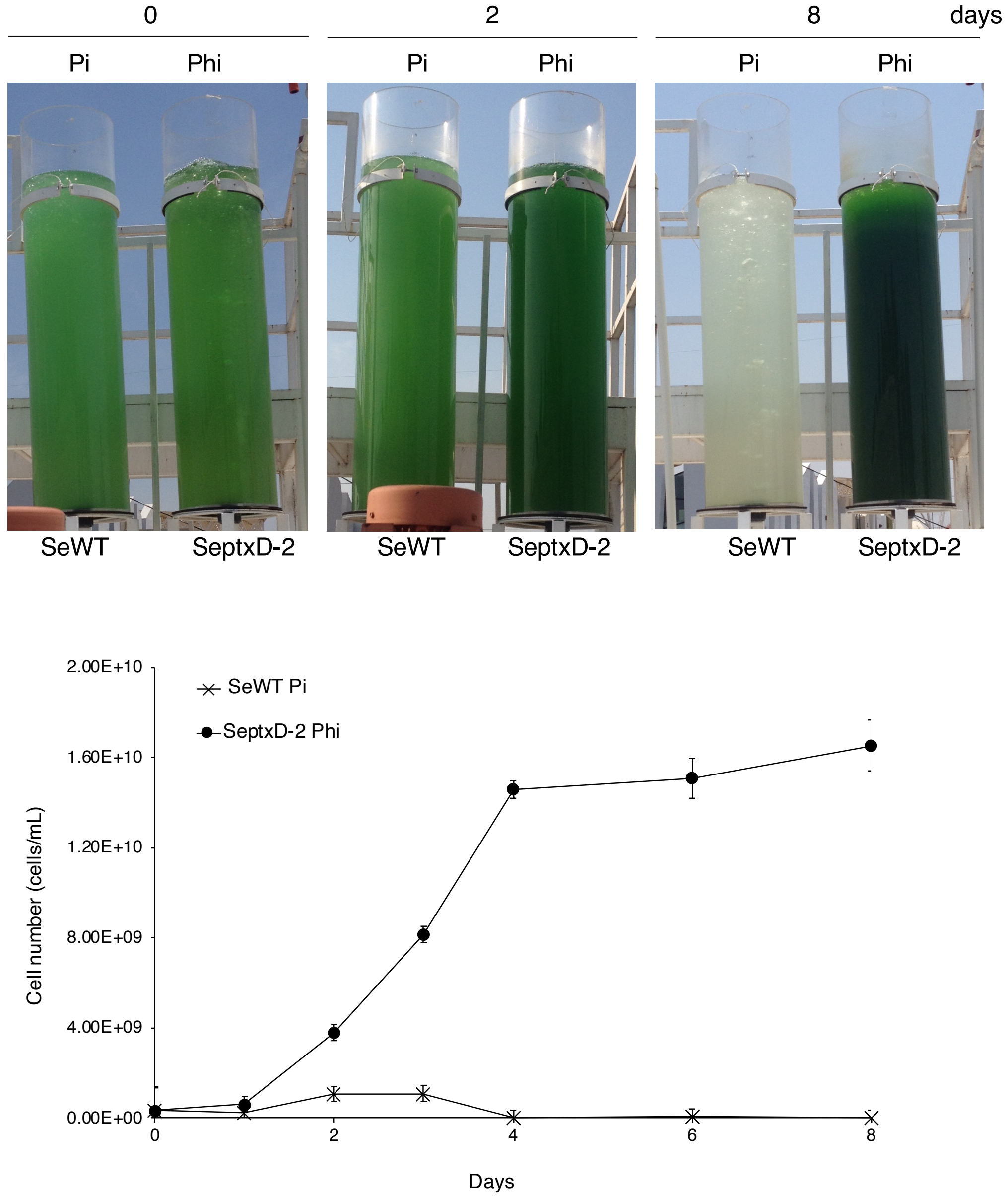

Figure S11. Outdoor growth of SeptxD-2 in 100 L cylindrical reactors using phosphite as phosphorus source under non-sterile media. a) *S. elongatus* transgenic strain (SeptxD-2) and wild type *S. elongatus* PCC 7942 (SeWT) were grown in BG-11 media prepared with industrial grade reagents and supplemented with 0.2 mM phosphate (Pi) or 1.8 mM phosphite (Phi) as P source. Cultures were performed using 7 % (v/v) inoculum and non-sterile 100 L cylindrical reactors bubbled with air. b) Cell number (cells/mL) was regularly determined throughout the eight days of the experiments. Data shown are the average of three replicates ± SD.

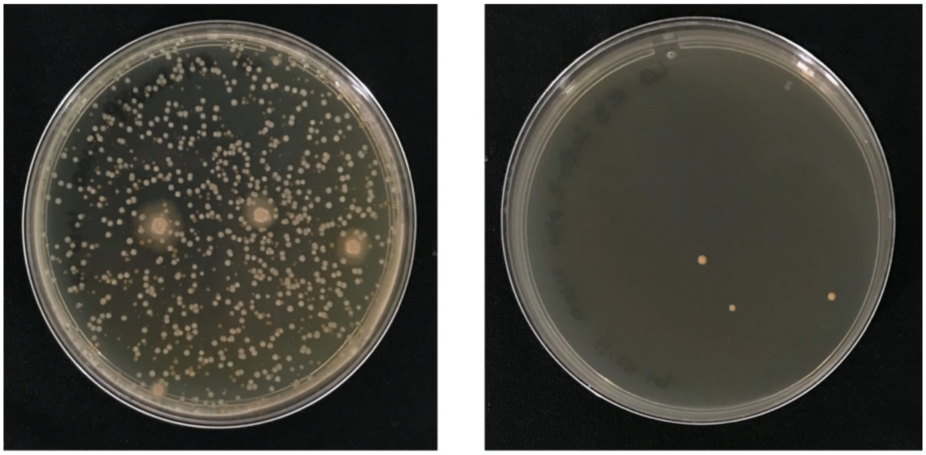

SeWT Pi   SeptxD-2 Phi

Figure S12. Bacterial contamination derived from SeptxD-2 and SeWT cultures outdoor. Pictures of Petri dishes in which Colony Forming Units (CFU) of samples from SeWT cultivated in phosphate medium (Pi) media and SeptxD-2 cultivated in phosphite medium (Phi) media, using 100 L cylindrical outdoor reactors, were determined.

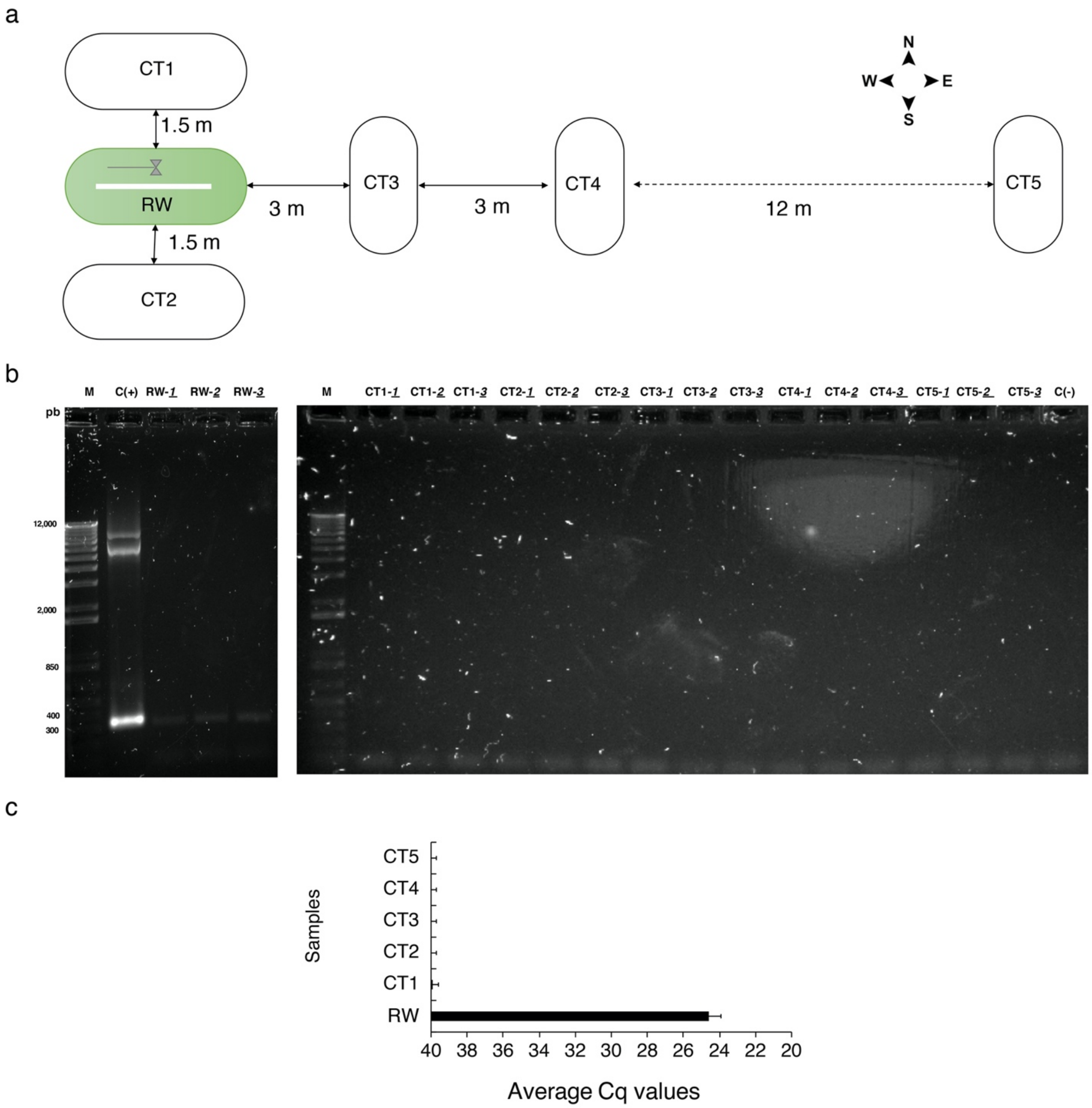

Figure S13. Assessment of the dispersion of SeptxD-2 during outdoor cultivation. a) Distribution of containers (CT1, CT2, CT3, CT4, CT5) used as traps during cultivation of SeptxD-2 in raceway pond (RW) outdoor. b) *ptxD* gene amplification by PCR in samples from the different traps (CT1-5) and the RW, using primers specific for the codon-optimized *ptxD* sequence. C(+): psyn_6_PtxDopt plasmid, positive control; C(-): water, negative control. Expected fragment is 304 bp. PCRs were performed in triplicate (1, 2, 3). M: DNA ladder marker. c) Real time-PCR to detect the *ptxD* gene in the same samples analyzed by PCR on (b).

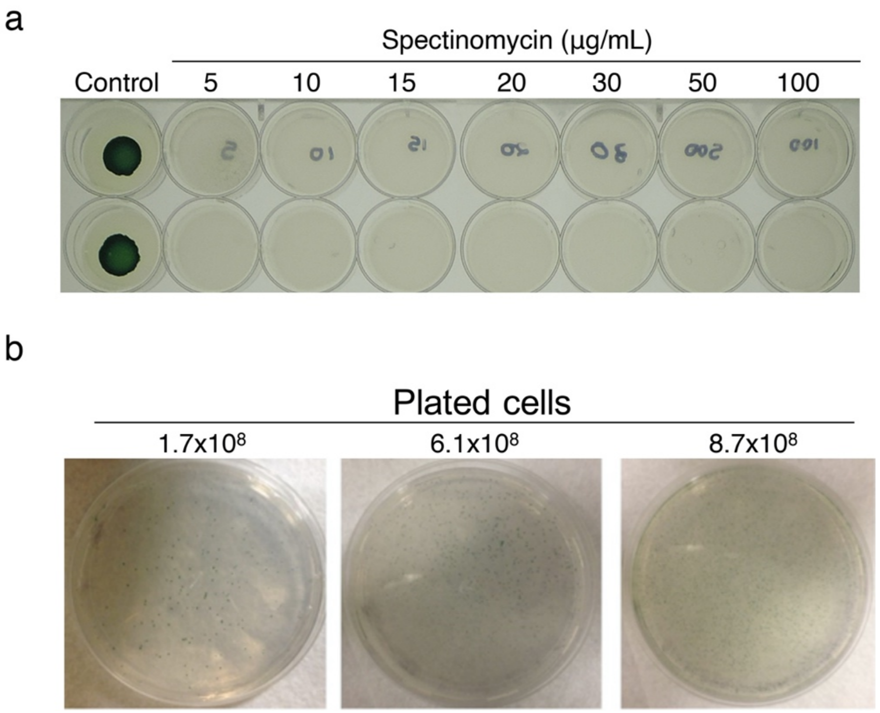

Figure S14. Selection of transformed *S. elongatus* using spectinomycin. a) Effect of different concentrations of spectinomycin on *S. elongatus* PCC 7942 growth in liquid media. B) After *S. elongatus* PCC 7942 recombination with the *psbAI::SeptxD* construct, three different amounts of cells (1.7, 6.1, and 8.7 x 10^8) were spread onto agar plates with BG-11 medium with 100 µg/mL spectinomycin. Photographs of the agar and multiwell plates were taken 15 days after cells were spread.

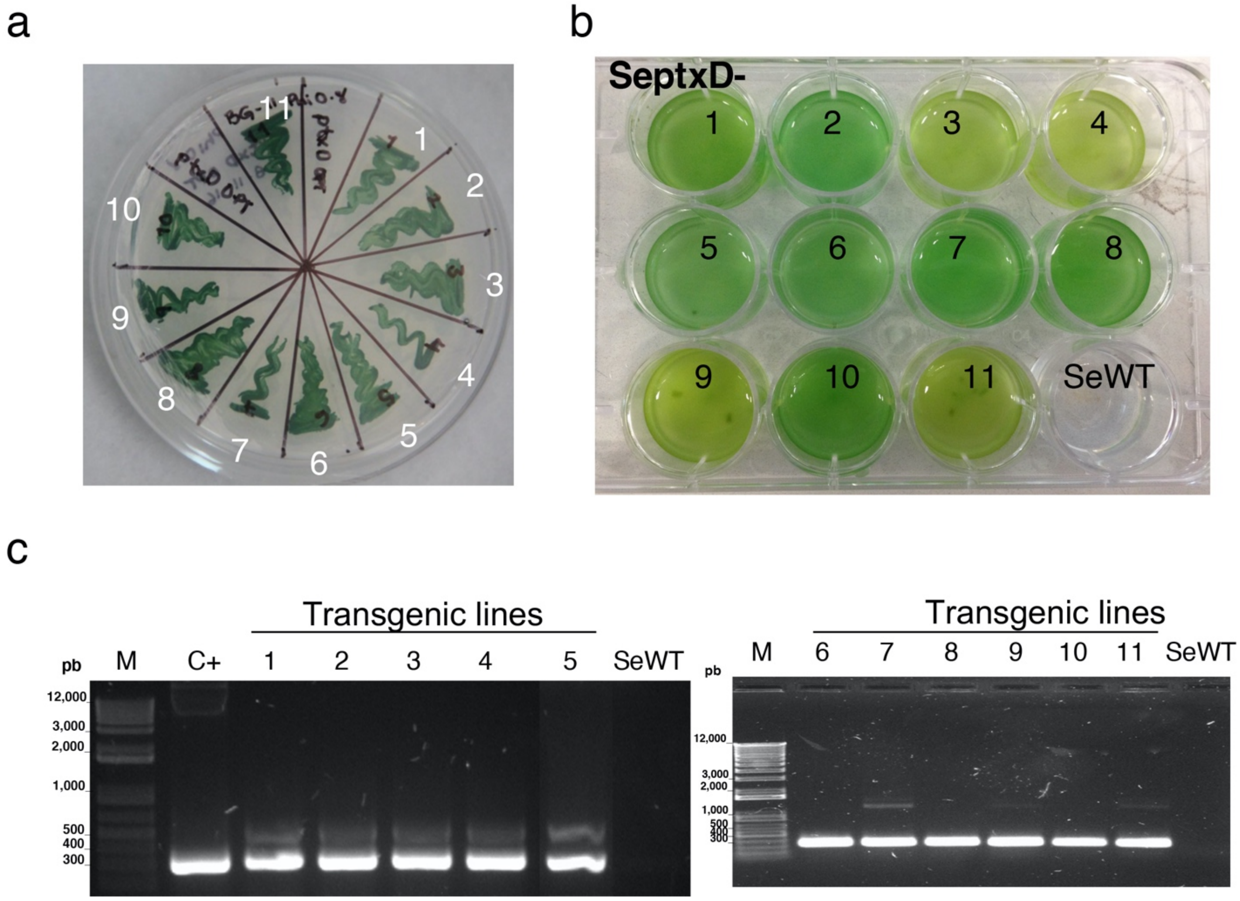

Figure S15. Independent colonies selected with the PtxD/phosphite system. Eleven isolated colonies obtained using the PtxD/phosphite system were cultured in agar plates with BG-11 media with 0.8 mM phosphite (a) and in liquid media with 1.8 mM Phi (b) as the sole phosphorus source. c) Colony PCR to verify the presence of the ptxD gene in the transgenic strains. SeWT: wild type; M: 1kb plus ladder (Invitrogen), C(+): positive control, plasmid.

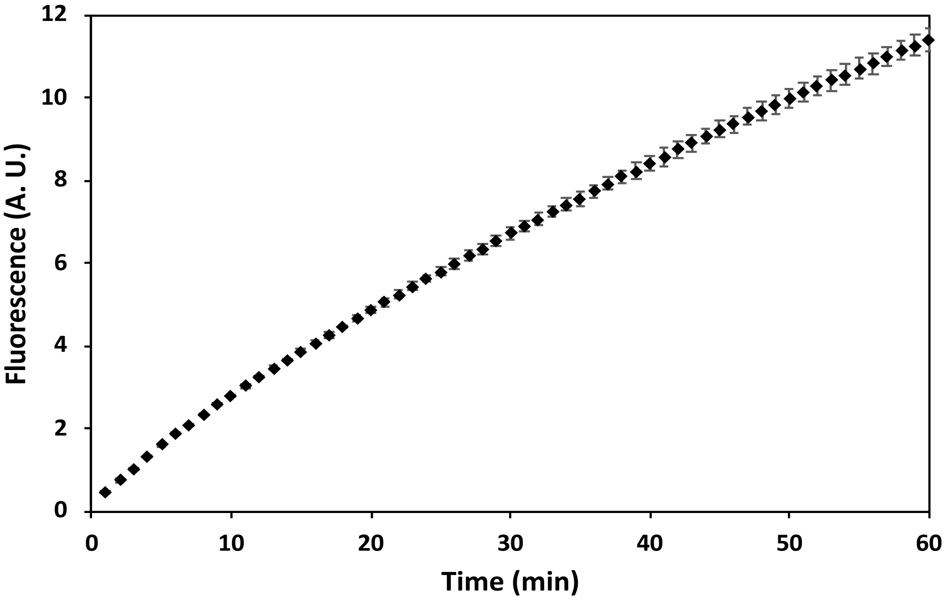

Figure S16. PtxD enzymatic activity expressed as Arbitrary Units of Fluorescence determined in SeptxD-2. The enzymatic activity was assayed in 50 mM MOPS, pH 7.25, 0.5 mM NAD^+, 1 mM phosphite, and a total desalted protein concentration of 50 μg. The reaction was incubated at 30 °C for 1 h. Values are means ± SE (n = 4).
