# Supplementary material for: Metabolic engineering of phosphite metabolism in Synechococcus elongatus PCC 7942 as an effective measure to control biological contaminants in outdoor raceway ponds
Source: Biotechnol Biofuels. 2020 Jul 9;13:119. doi: 10.1186/s13068-020-01759-z (PMC7346359; doi:10.1186/s13068-020-01759-z)
Supplement: Supplementary file 2 — Additional file 2. Additional tables and legends supporting the results described in the text. [file 13068_2020_1759_MOESM2_ESM.pdf]

Additional file 2

Metabolic engineering of phosphite metabolism in *Synechococcus elongatus* PCC 7942 as an effective measure to control biological contaminants in outdoor raceway ponds

Sandra Isabel González-Morales[1], Navid Pacheco-Gutiérrez[1], Carlos A. Ramírez-Rodríguez[1], Alethia A. Brito-Bello[1], Priscila Estrella-Hernández[1], Luis Herrera-Estrella[2,3], Damar L. López-Arredondo[1,3,§]

[1]StelaGenomics México, S de RL de CV, Av. Camino Real de Guanajuato s/n, 36821 Irapuato, Guanajuato, Mexico.

[2]Laboratorio Nacional de Genómica para la Biodiversidad, Unidad de Genómica Avanzada del Centro de Investigación y de Estudios Avanzados del Instituto Politécnico Nacional, Km 9.6 carretera Irapuato León, Irapuato, 36500 Guanajuato, Mexico.

[3]Institute of Genomics for Crop Abiotic Stress Tolerance, Texas Tech University, Lubbock, TX, 79409 USA.

[§]To whom correspondence should be addressed:

Damar López-Arredondo, email: Damar.Lopez-Arredondo@ttu.edu; Tel. 806 8343364, orcid: https://orcid.org/0000-0001-7389-3143

Table S1. Identification of the consortium Comp 1 based on 16S rRNA sequencing.

Sample	Primers	Best hit	Nucleotide Blast description	E value	Grade†	Bit-Score‡
**Comp1**	**1492R**	1	*Nostoc sp.* Bahar_M 16S ribosomal RNA gene, partial sequence	0	98.5	1,836.69
		2	*Nostoc sp.* FSN-E 16S ribosomal RNA gene, partial sequence	0	98.5	1,836.69
		3	*Nostoc sp.* Bahar_E 16S ribosomal RNA gene, complete sequence	0	98.5	1,836.69
**Comp1**	**27F**	1	*Aliinostoc sp.* SA43 16S ribosomal RNA gene and 16S-23S ribosomal RNA intergenic spacer, partial sequence	0	98.7	1,929.02
		2	*Aliinostoc sp.* SA43 16S ribosomal RNA gene and 16S-23S ribosomal RNA intergenic spacer, partial sequence	0	98.7	1,929.02
		3	*Nostoc sp.* CENA88 16S ribosomal RNA gene, partial sequence	0	98.7	1,923.48
**Comp1**	**907R**	1	*Nostoc sp.* CENA543 chromosome, complete genome	0	83.0	1,447.05
		2	*Nostoc sp.* CENA543 chromosome, complete genome	0	83.0	1,447.05
		3	*Aliinostoc sp.* SA43 16S ribosomal RNA gene and 16S-23S ribosomal RNA intergenic spacer, partial sequence	0	82.8	1,432.27

†A weighted score for the hit comprised of the E value, the pairwise identity and the coverage.
‡ Bit-score for the hit.

Table S2. Example of outdoor conditions during the experiments with 100-L bioreactors in the summer, 2017 in Irapuato, Guanajuato, México.

DAI	Environmental temperature (°C)		SeptxD-2 (Phi)			WT (Pi)			Solar irradiance (kW h m^{-2})
			Culture temperature (°C)		pH	Culture temperature (°C)		pH	
	Minimum	Maximum	Minimum	Maximum		Minimum	Maximum		
0	18	29	21	33	7	nd	nd	7.0	7.3
1	19	32	23	35	6.7	nd	nd	7.2	7.3
2	19	33	24	35	6.5	nd	nd	7.2	7.0
3	19	32	24	34	6.6	nd	nd	7.1	7.0
4	20	32	25	35	6.6	nd	nd	7.0	7.0
5	20	30	26	32	6.5	nd	nd	6.8	6.8
6	22	27	27	30	6.9	nd	nd	6.7	6.8
7	20	32	26	35	6.6	nd	nd	6.8	6.8

Pi: phosphate; Phi, phosphite; DAI: days after inoculation; nd: no data.

Table S3. Example of outdoor conditions during the experiments with 100-L bioreactors in the fall, 2016 in Irapuato, Guanajuato, México.

DAI	Environmental temperature (°C)		SeptxD-2 (Phi)			WT (Pi)			Solar irradiance (kW h m^{-2})
			Culture temperature (°C)		pH	Culture temperature (°C)		pH	
	Minimum	Maximum	Minimum	Maximum		Minimum	Maximum		
0	10	28	15	31	6.5	nd	nd	6.3	5.7
1	13	25	15	29	6.1	nd	nd	6.2	5.7
2	15	20	19	25	6.6	nd	nd	6.2	5.7
3	12	26	16	30	6.7	nd	nd	6.2	5.6
4	12	26	15	31	6.8	nd	nd	6.4	5.6
5	14	28	17	31	7.0	nd	nd	6.0	5.6
6	12	30	15	34	6.9	nd	nd	6.2	5.6
7	13	29	16	32	6.9	nd	nd	6.1	5.6

Pi: phosphate; Phi, phosphite; DAI: days after inoculation; nd: no data.

Table S4. Example of outdoor conditions during the experiments with 1000-L open ponds in the summer, 2019 in Irapuato, Guanajuato, México.

DAI	Environmental temperature (°C)		SeptxD-2 (Phi)			WT (Pi)			Solar irradiance (kW h m^{-2})
			Culture temperature (°C)		pH	Culture temperature (°C)		pH	
	Minimum	Maximum	Minimum	Maximum		Minimum	Maximum		
0	17	31	16	30	7	nd	nd	7.2	7.2
1	19	32	19	31	6.8	nd	nd	7	7.2
2	16	33	16	32	6.8	nd	nd	7	7.0
3	16	31	15	31	6.8	nd	nd	6.9	7.0
4	17	30	16	30	6.9	nd	nd	6.9	7.0
5	19	29	19	29	6.9	nd	nd	6.9	7.0
6	17	28	16	29	6.7	nd	nd	6.9	6.8
7	15	31	15	30	6.7	nd	nd	6.9	6.8

Pi: phosphate; Phi, phosphite; DAI: days after inoculation; nd: no data.

Table S5. Composition and cost of BG-11 media using industrial grade reagent.

BG-11 medium	Amount per L of medium (Kg)	Bulk price[1] (\$/Kg)	Price per 1,000 L of medium (\$)		
			Pi as P source	Phi as P source	Pi as P source with antibiotic
$NaNO_3$	0.0015	0.50	0.75	0.75	0.75
K_2HPO_4 [3]	0.00004002	2.00	0.08	0	0.08
K_2HPO_3 [3]	0.000216	1.38	0	0.30	0
$MgSO_4 \cdot 7H_2O$	0.000074	0.15	0.01	0.01	0.01
$CaCl_2 \cdot 2H_2O$	0.000035	0.20	0.01	0.01	0.01
Citric Acid	0.000006	0.60	0.004	0.004	0.004
Ferric Ammonium Citrate	0.0000055	30.00	0.17	0.17	0.17
$Na_2EDTA \cdot 2H_2O$	0.000001	2.00	0.002	0.002	0.002
Na_2CO_3	0.00002	0.26	0.01	0.75	0.01
Kanamycin sulfate [2]	0.0001	150	-	-	1.50
Total			**1.02**	**1.24**	**2.52**

[1] Minimum price based on Alibaba.com, 2020.

[2] Use of Kanamycin sulfate as antibiotic to control contamination is calculated considering doses reported by (Jaiswal, et al., 2018).

[3] Concentration of phosphate (Pi) and phosphite (Phi) in growth media is 0.2 and 1.8 mM, respectively.

Table S6. Estimated cost to operate cylindrical bioreactors (100 L) and open ponds (1,000 L) of a total capacity of 3,300 L.

System	Capital cost ($)[1]				Variable operating cost ($)					Total operating cost ($)	Total cost per Kg of biomass ($)
	Cylindrical reactors	Raceway reactors	Filtration station	Depreciation[2]	Medium culture[4]	Energy[3]	CP[3]	Water[3]	Medium filters[3]		
**Phosphate + sterilization by filtration**	7,261.88	12,790.02	3,425.00	8.38	3.00	9.91	0.01	1.83	11.11	**34.24**	**32.61**
**Phosphate + antibiotic**	7,261.88	12,790.02	0.0	6.08	7.47	9.70	0.01	1.83	0.0	**25.13**	**23.89**
**Only Phosphite**	7,261.88	12,790.02	0.0	6.08	3.63	9.70	0.01	1.83	0.0	**21.24**	**20.23**

[1] Capital costs were estimated for a small pilot plant with operational capacity of 3,300 L, composed of three cylindrical bioreactors (100 L) and three open ponds (1,000L). Vendor quotations from "Servo Soluciones Industriales S.A de C.V" (2020) for the pilot plant and the filtration station for a total operational capacity of 3,500 L were used as a basis for equipment calculation. Cylindrical reactor cost includes acrylic reactor, blower for air supply to cylindrical bioreactors, hydraulic devices (tubes and valves manufactured with PVC material), electrical devices and installation. Raceway cost includes civil engineering, hydraulic devices (tubes and valves manufactured with PVC material), electrical infrastructure (motors, control cabinet, and cabling), stainless-steel propeller devices and installation. Filtration station cost includes pumps to move the media and tanks to store the media for recirculation into the system.

[2] To convert the calculation of capital cost into variable cost, a culture cycle of 12 days for phosphate medium (with sterilization by filtration), and 10 days for phosphate media with antibiotics medium and phosphite-based media, was considered. The raceway reactor was assumed to operate 24 hours per day for 330 days per year; performing 28 batches per year for the phosphate medium (with sterilization by filtration) system; 33 batches per year for phosphate with antibiotics and phosphite-based media systems. Depreciation was calculated considering 10 years life time for the pilot plant with an annual depreciation cost 10 % of capital cost. The depreciation was calculated with the formula Depreciation= Cost of Asset – Salvage Value/Useful Life of Asset.

[3] Energy cost to operate the pilot plant was estimated considering: one blower of 2 HP capacity, with energy consumption of 126 KWH by 9 days of operation, 3 motors of 1.5 HP capacity, with energy consumption of 151.2 KWH by 9 days of operation, and 2 pumps for the filtration system of 0.5 HP capacity, with energy consumption of 6 KWH. The Cleaning Process (CDP) to avoid cross contamination between transgenic lines was performed using a solution of calcium hypochlorite at 1.5 mg/L to clean the reactor and piping. Water cost was considered the industrial service fee from drinking water supply and sanitation services on Irapuato, Guanajuato, Mexico. The cost of medium filter was of $100 with life utilization of 3 months.

[4]Growt media preparation was considered according prices in Supplementary Table 5. Cylindrical bioreactors and raceways ponds operated using 7 and 10 % inoculum, respectively. The total cost per Kg of biomass was calculated considering total operating cost and 0.35 g/L as yield of biomass.
